# Gene-Specific Intron Retention Serves as Molecular Signature that Distinguishes Melanoma from Non-Melanoma Cancer Cells in Greek Patients

**DOI:** 10.3390/ijms20040937

**Published:** 2019-02-21

**Authors:** Aikaterini F. Giannopoulou, Eumorphia G. Konstantakou, Athanassios D. Velentzas, Socratis N. Avgeris, Margaritis Avgeris, Nikos C. Papandreou, Ilianna Zoi, Vicky Filippa, Stamatia Katarachia, Antonis D. Lampidonis, Anastasia Prombona, Popi Syntichaki, Christina Piperi, Efthimia K. Basdra, Vassiliki Iconomidou, Evangelia Papadavid, Ema Anastasiadou, Issidora S. Papassideri, Athanasios G. Papavassiliou, Gerassimos E. Voutsinas, Andreas Scorilas, Dimitrios J. Stravopodis

**Affiliations:** 1 Section of Cell Biology and Biophysics, Department of Biology, School of Science, National and Kapodistrian University of Athens, 15701 Athens, Greece; Katia_13g@hotmail.com (A.F.G.); EKONSTANTAKOU@mgh.harvard.edu (E.G.K.); tveletz@biol.uoa.gr (A.D.V.); npapand@biol.uoa.gr (N.C.P.); skatarachia@biol.uoa.gr (S.K.); labant@aua.gr (A.D.L.); veconom@biol.uoa.gr (V.I.); ipapasid@biol.uoa.gr (I.S.P.); 2 Laboratory of Molecular Carcinogenesis and Rare Disease Genetics, Institute of Biosciences and Applications, National Center for Scientific Research “Demokritos”, 15310 Athens, Greece; savgeris@bio.demokritos.gr (S.N.A.); mvoutsin@bio.demokritos.gr (G.E.V.); 3 Section of Biochemistry and Molecular Biology, Department of Biology, School of Science, National and Kapodistrian University of Athens, 15701 Athens, Greece; margaritis.avgeris@gmail.com (M.A.); ascorilas@biol.uoa.gr (A.S.); 4 Department of Biological Chemistry, Medical School, National and Kapodistrian University of Athens, 11527 Athens, Greece; ilianna.zoi@gmail.com (I.Z.); cpiperi@otenet.gr (C.P.); ebasdra@med.uoa.gr (E.K.B.); papavas@med.uoa.gr (A.G.P.); 5 Center of Basic Research, Biomedical Research Foundation of the Academy of Athens, 11527 Athens, Greece; vickyrougefilippa@gmail.com (V.F.); synticha@bioacademy.gr (P.S.); anastasiadou@bioacademy.gr (E.A.); 6 Laboratory of Chronobiology, Institute of Biosciences and Applications, National Center for Scientific Research “Demokritos”, 15310 Athens, Greece; prombona@bio.demokritos.gr; 7 2nd Department of Dermatology and Venereology, Medical School, National and Kapodistrian University of Athens, “Attikon” University Hospital, 12462 Athens, Greece; epapad@med.uoa.gr

**Keywords:** BCC, cancer, intron, melanoma, SCC, splicing, transcription

## Abstract

Background: Skin cancer represents the most common human malignancy, and it includes BCC, SCC, and melanoma. Since melanoma is one of the most aggressive types of cancer, we have herein attempted to develop a gene-specific intron retention signature that can distinguish BCC and SCC from melanoma biopsy tumors. Methods: Intron retention events were examined through RT-sqPCR protocols, using total RNA preparations derived from BCC, SCC, and melanoma Greek biopsy specimens. Intron-hosted miRNA species and their target transcripts were predicted via the miRbase and miRDB bioinformatics platforms, respectively. Ιntronic ORFs were recognized through the ORF Finder application. Generation and visualization of protein interactomes were achieved by the IntAct and Cytoscape softwares, while tertiary protein structures were produced by using the I-TASSER online server. Results: *c-MYC* and *Sestrin-1* genes proved to undergo intron retention specifically in melanoma. Interaction maps of proteins encoded by genes being potentially targeted by retained intron-accommodated miRNAs were generated and *SRPX2* was additionally delivered to our melanoma-specific signature. Novel ORFs were identified in *MCT4* and *Sestrin-1* introns, with potentially critical roles in melanoma development. Conclusions: The property of *c-MYC*, *Sestrin-1*, and *SRPX2* genes to retain specific introns could be clinically used to molecularly differentiate non-melanoma from melanoma tumors.

## 1. Introduction

Human epidermis contains ~1.5 × 10^2^ melanocytes per square millimeter, which corresponds to ~3 × 10^9^ cells in the skin of an average man. They divide less than twice a year and their main function is to provide melanin to adjacent keratinocytes, in order to protect them from the harmful effects of UV radiation. Cutaneous response to UV radiation exposure involves a DNA damage-driven activation of p53 protein in keratinocytes. As a result, they synthesize and secrete α-MSH that stimulates MC1R on neighboring melanocytes to induce synthesis of melanin pigment, which is next delivered back to keratinocytes in the form of melanosomes [1,2,3].

Cutaneous cancer, including BCC, SCC, and melanoma [4], represents a major public health issue due to its high and rising incidence, substantial mortality, elevated medical cost, and treatment-associated deformity [5]. BCC and SCC derive from skin epidermis and affect different layers of its stratified structure, whereas melanoma arises from malignant transformation of melanocytes [3,6]. BCC grows slowly, invade locally, metastasize rarely and comprises ~75% of non-melanoma skin cancer cases. In contrast, SCC grows more rapidly and invades and metastasizes more frequently (~2–3%), and accounts for ~20% of non-melanoma skin cancer cases [6,7,8]. However, although malignant melanoma represents only ~4% of all skin cancers, it usually becomes highly invasive and metastatic, genetically heterogeneous (with high mutational load), and therapeutically refractory [1,3,6].

Aberrant Hedgehog signaling that is directed by mutations in the *PTCH1*, *SMO*, and *SUFU* pathway genes serves as a pivotal defect causing BCC formation. Additional driver mutations are identified in the cancer-related genes *MYCN*, *LATS1*, *PIK3CA*, and *RAS* family members. Interestingly, specific expression of *CASP8* splice variants that retain sequences from intron 8, presumably offering apoptosis resistance, seems to also represent another BCC molecular signature [9,10,11]. Regarding SCC pathogenesis, a number of genetic determinants have been previously reported, including the pigmentation genes *MC1R*, *OCA2*, and *TYR*, the kinetochore-associated gene *KNSTRN*, the transcription regulation gene *SOX2*, and the signal transduction genes *TGFBR1* and *TGFBR2* [12,13,14,15]. Other high-risk mutations in the tumor-regulating genes *TP53*, *CDKN2A*, *HRAS*, and *NOTCH1/2* are also implicated in the disease [16,17,18]. Genome landscapes of major melanoma subtypes are heavily mutated and harbor critical genetic alterations in several genes controlling cancer initiation and progression, such as the *BRAF*, *NRAS*, *NF1*, *TP53*, and *CDKN2A* ones, thus conferring subtype-dependent mutagen signatures and therapeutic targets on melanoma [1,19,20]. Disease mutation burden is also increased by aberrations in several other genes, with the melanocyte lineage-specific oncogene *MITF* being one of the characteristic examples [19,21,22,23]. Moreover, promoter mutations in the telomere-related gene *TERT* critically contribute to the familial and sporadic forms of human melanoma, therefore indicating the important role of noncoding sequences in skin cancer development [1,19,20,24,25].

Irregularities in the splicing machinery may create novel vulnerabilities in tumor cells that can be therapeutically exploited. Somatic mutations in genes encoding spliceosomal proteins and RNA factors are detected at high frequency in a number of cancers, including uveal melanoma [26]. Abnormal RNA splicing can be typified by widespread intron retention, even in the absence of mutations affecting the splicing process [27]. Interestingly, large-scale transcriptome profiling has shown widespread intron retention in malignant tissues. For example, 2038 and 2340 intron retention events have been detected in breast cancer and lung carcinoma, respectively, while intron 4 of *KLK3* (*PSA*) transcript was specifically retained in the majority of patients in a prostate cancer cohort [28,29,30,31]. Notably, intron retention has been previously reported to serve as a common pathogenic mechanism of tumor-suppressor inactivation [32]. Hence, intron-containing mRNAs seem to contribute to the transcriptional diversity of human cancers, likely modulating their malignant character and response to therapy.

Therefore, we herein attempted to investigate the association of intron retention occurrence with BCC, SCC, and melanoma development in Greek patients. Surprisingly, our results reveal a novel molecular signature of gene-specific intron retention in melanoma, but not in BCC and SCC biopsies, with new intron-encoded, putative proteins likely acting as melanoma regulators, and thus promising drug targets for the disease.

## 2. Results

### 2.1. Melanoma-Specific Intron Retention of c-MYC Gene

**Figure 1:**
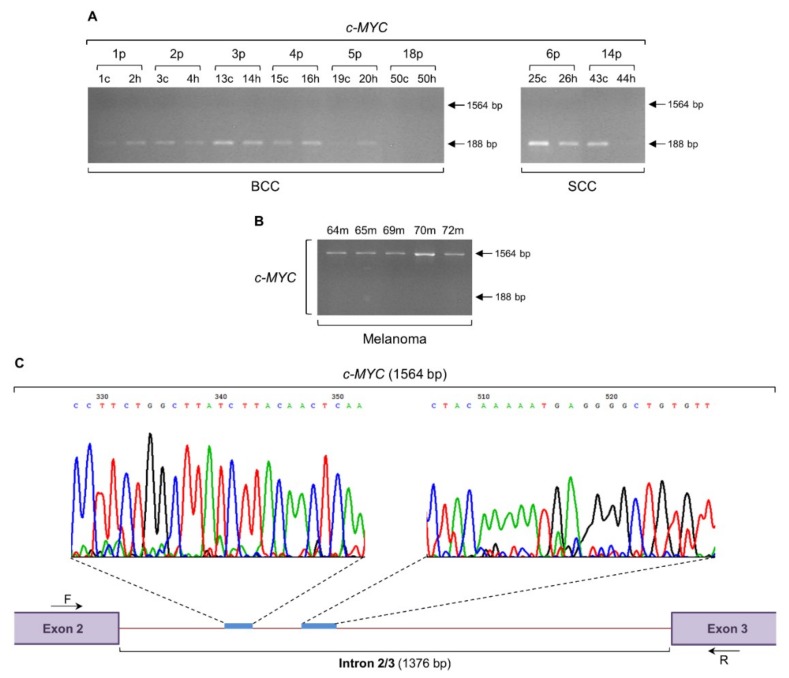
The *c-MYC* gene is subjected to an intron retention mechanism in human melanoma. Gene expression profiles of *c-MYC*, via employment of RT-sqPCR protocols, using total RNA preparations derived from BCC and SCC (**A**) or melanoma (**B**) biopsy specimens. *GAPDH* served as gene of reference (also, see Figure 6F and Table S1). (**C**) Representative DNA chromatogram derived from cycle sequencing of the melanoma-specific 1564 bp long PCR product. Characteristic *c-MYC* intron 2/3 (1376 bp) sequenced areas are indicated. Besides intron 2/3 retention (1564 bp), note the absence of *c-MYC* normal transcriptional activity (lack of 188 bp) in all melanoma cDNA preparations (**B**). p: patient, c: cancer tissue (biopsy), h: healthy tissue (biopsy), m: melanoma (biopsy), bp: base pair, F: forward (primer) and R: reverse (primer).

Spliceosome has been previously shown to serve as a novel target of oncogenic stress in c-MYC-dependent cancers, with perturbation of spliceosome in c-MYC-hyperactivated cells resulting in global intron retention [33]. Notably, c-MYC seems to maintain the splicing fidelity of exons (with a weak 5′ donor site), while depletion of c-MYC in lymphoma cells is associated with an aberrant splicing process [34]. Given c-MYC’s major role in transcript maturation, its central position as a molecular driver in melanoma development [35,36], and the ability to confer resistance to chemotherapy [37], we herein examined the transcriptional expression profile of *c-MYC* gene in BCC, SCC, and melanoma biopsy specimens. The expected PCR product of 188 bp long was detected exclusively in BCC and SCC groups (Figure 1A), whereas a higher molecular weight fragment of 1564 bp long could be specifically recognized in all melanoma samples (Figure 1B). Purification and cycle sequencing of the 1564 bp band, and comparison of the obtained DNA sequence with the respective reference one derived from Ensembl Genome Browser 91 bioinformatics platform, unveiled the retention of a 1376 bp long DNA segment that represents the intron 2/3 of *c-MYC* gene (ENST00000621592.5) (Figure 1C). Altogether, it seems that the *c-MYC* intron retention, together with the lack of *c-MYC* legitimate transcriptional activity, can serve as a novel and powerful biomarker for human melanoma versus BCC and SCC skin cancers.

### 2.2. The MCT4 Gene is Subjected to an Intron Retention Process both in Non-Melanoma and Melanoma Biopsy Specimens

**Figure 2:**
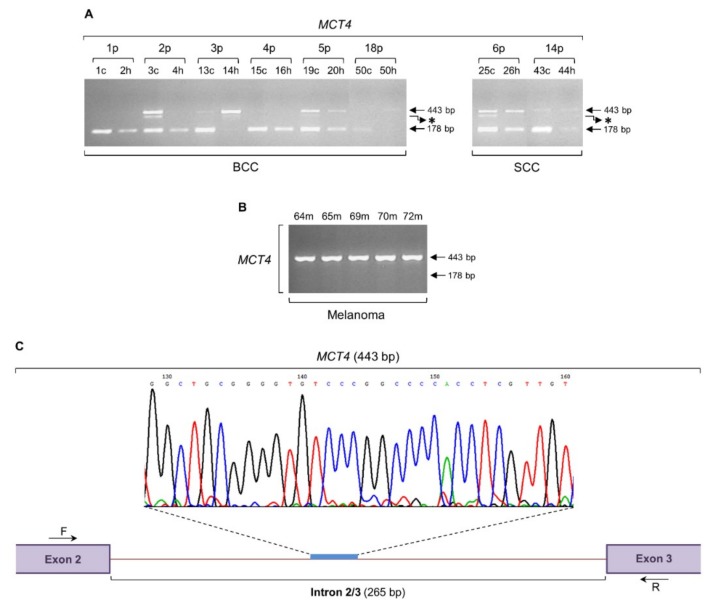
*MCT4* intron retention is activated both in non-melanoma (BCC and SCC) and melanoma biopsy collections. Patterns of *MCT4* transcriptional activity, through engagement of RT-sqPCR platforms based on total RNA extraction from BCC and SCC (**A**), or melanoma (**B**) biopsy samples. *GAPDH* was used as control gene (also, see Figure 6F and Table S1). (**C**) Representative DNA sequence chromatogram of the melanoma-specific 443 bp long PCR fragment. A characteristic *MCT4* intron 2/3 (265 bp) sequenced area is indicated. Besides the retention of *MCT4* intron 2/3 (443 bp), note the absence of gene’s normal transcriptional activity (lack of 178 bp) in all melanoma cDNA samples (B). p: patient, c: cancer tissue (biopsy), h: healthy tissue (biopsy), m: melanoma (biopsy), bp: base pair, F: forward (primer), R: reverse (primer) and asterisk (*): aberrant splicing-derived PCR band.

Since a number of metabolic pathways are promoted by c-MYC [38,39], and metabolic rewiring is required for development of melanoma and its response to therapy [40], we next investigated the phenomenon of intron retention in a gene of the MCT (monocarboxylate transporter) family (Figure 2), whose critical *MCT1* (*SLC16A1*) gene member is a target and can be directly activated by c-MYC [41,42]. The MCT4 (SLC16A3) protein catalyzes the proton-linked bidirectional transport of monocarboxylates, such as lactate, pyruvate, and ketone bodies, across the cell membrane [43], and its elevated expression is associated with progression to advanced melanomas [44,45]. In contrast to *c-MYC* (Figure 1), the *MCT4* gene (ENST00000581287.5) is herein presented to undergo an intron retention process not only in melanoma but also in BCC and SCC patients (Figure 2). Surprisingly, besides the expected PCR product of 178 bp long, all three types of skin cancer carry an additional band of 443 bp long that is detected in both cancer (“c”, or “m”) and healthy (“h”) tissue specimens (Figure 2A,B). Cycle sequencing of the melanoma-specific 443 bp purified PCR product and subsequent comparison of the obtained DNA sequence with the reference one extracted from Ensembl Genome Browser 91 revealed the retention of intron 2/3 (265 bp) within the 443 bp DNA segment (Figure 2C). Intriguingly, a third PCR band that migrates slightly lower than the 443 bp one seems to be preferentially expressed in BCC and SCC cancer samples, thus dictating the non-melanoma-dependent activation of additional *MCT4* aberrant splicing events (Figure 2A,B). Despite the inability of *MCT4* intron retention to be used alone as molecular signature to distinguish melanoma from BCC and SCC cancers (Figure 2A,B), the *MCT4* transcriptional upregulation in BCC and SCC cancer versus healthy matched tissues, as indicated by the amplification profiles of 178 bp PCR fragment (Figure 2A), could be successfully exploited as a powerful BCC and SCC diagnostic marker for each disease.

### 2.3. Intron Retention of Sestrin-1 Gene can Distinguish Non-Melanoma from Melanoma Tissue

**Figure 3:**
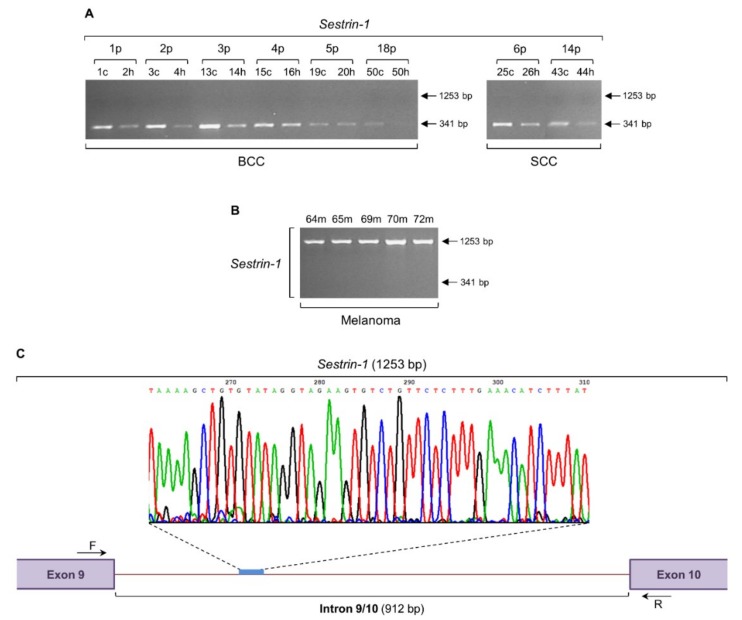
Intron retention of *Sestrin-1* gene molecularly differentiates BCC and SCC from melanoma cells. Expression profiles of *Sestrin-1* gene, via employment of RT-sqPCR protocols and utilization of total RNA preparations purified from BCC and SCC (**A**) or melanoma (**B**) biopsy collections. *GAPDH* served as gene of reference (also, see Figure 6F and Table S1). (**C**) Representative DNA chromatogram derived from cycle sequencing of the melanoma-specific 1253 bp long PCR band. A characteristic *Sestrin-1* intron 9/10 (912 bp) sequenced area is indicated. Besides intron 9/10 retention (1253 bp), note the absence of *Sestrin-1* normal transcriptional activity (lack of 341 bp) in all melanoma cDNA preparations (B). p: patient, c: cancer tissue (biopsy), h: healthy tissue (biopsy), m: melanoma (biopsy), bp: base pair, F: forward (primer) and R: reverse (primer).

The evidence linking activated mTOR-network signaling to human cancer has generated great interest in biomarker profiling and therapeutic targeting. Importantly, it has been reported that nonsynonymous mTOR mutations are frequent and likely predict a worse prognosis in melanoma patients [46,47]. The proficiency of Sestrins to tightly control mTOR signaling, which is normally implicated in survival, growth, proliferation, translation, metabolism, autophagy, and cytoskeletal organization in mammalian cells [47,48,49,50], prompted us to, next, examine the *Sestrin-1* (*SESN1*) gene for presumable events of intron retention (Figure 3). The expected 341 bp long PCR product could be detected in both BCC and SCC patient groups (Figure 3A). However, instead of the 341 bp DNA segment, a higher molecular weight, strong intensity (as compared to the hardly detected one in a few BCC and SCC samples) PCR fragment of 1253 bp long was obtained for all melanoma specimens (Figure 3B). Purification and cycle sequencing of the melanoma-derived 1253 bp PCR band and suitable alignment of the unveiled sequence with the reference one extracted from the Ensembl Genome Browser 91 proved the presence of a 912 bp long DNA sequence that corresponds to the intron 9/10 (912 bp) of *Sestrin-1* gene (ENST00000436639.6) (Figure 3C). Conclusively, intron retention of *Sestrin-1* may serve as a novel and reliable biomarker with important clinical value for melanoma diagnosis and therapy monitoring. Furthermore, the strongly activated intron retention of *Sestrin-1* gene, together with the absence of its processed and normally spliced transcript, could significantly contribute to the genotypic distinction of melanoma from BCC and SCC skin cancers, and thus could be successfully adopted in the clinical management of the disease.

### 2.4. Mapping the miRNA Landscape of c-MYC, MCT4 and Sestrin-1 Retained Introns: from Target Genes to Protein Interactomes

**Figure 4:**
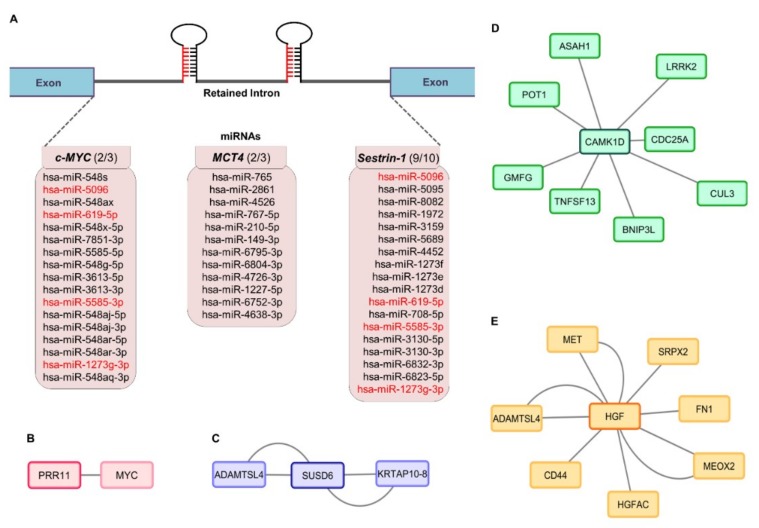
Retained intron-specific profiling of miRNA production: from miRNA target genes to their protein–protein interaction maps. (**A**) Collection of miRNA molecules in silico predicted, via miRbase employment, to align to the respective *c-MYC* (2/3), *MCT4* (2/3), and *Sestrin-1* (9/10) retained intron herein examined. (B–E) Molecular interactomes, through Intact and Cytoscape engagement, of proteins encoded by genes that share the property to be presumably targeted by at least three distinct miRNAs hosted within the *c-MYC* (2/3), *MCT4* (2/3), and *Sestrin-1* (9/10) retained introns (also, see Table 1 and Table S2). miRDB was the database used for miRNA target transcript identification. (**B**) PRR11 protein interactome (one member). (**C**) SUSD6 (KIAA0247) protein interactome (two members). (**D**) CAMK1D protein interactome (eight members). (**E**) HGF protein interactome (seven members). Fonts in red color denote the common miRNAs in between *c-MYC* (2/3) and *Sestrin-1* (9/10) retained introns (**A**).

In an effort to ascribe a presumable function to the gene-specific intron retention process herein identified, and given that approximately 50% of all human miRNAs reside within introns of coding genes [51], with certain intronic species (miR-211) critically modulating the malignant and invasive characteristics of melanoma [52], we assumed that the *c-MYC*, *MCT4*, and *Sestrin-1* retained introns could serve as sources of miRNA generation. miRNAs (microRNAs) represent a class of endogenous, small (~23 nucleotides long) noncoding RNA molecules that are generally transcribed by RNA polymerase II, and play central roles in cell survival, proliferation, and differentiation through specific binding to complementary target mRNAs, causing mRNA translational inhibition and/or degradation [53,54,55]. Interestingly, among the several miRNAs that are related to melanoma, miR-211 is hosted within an intron of a gene called *Melastatin*, and as such regulates the invasiveness of malignant cells [52,56]. Hence, by employing the miRbase bioinformatics tool, we recognized a number of miRNAs that could successfully align to each one of the three retained intronic sequences herein examined. Seventeen, twelve, and eighteen human (*Homo sapiens*: hsa) miRNA molecules were presented to carry homologous sequences with the ones of intron 2/3 for *c-MYC*, intron 2/3 for *MCT4*, and intron 9/10 for *Sestrin-1* genes, respectively (Figure 4A). Surprisingly, *c-MYC*- and *Sestrin-1*-retained introns proved to share four distinct miRNAs: miR-5585-3p, miR-1273g-3p, miR-619-5p, and miR-5096 (Table 1). Next, engagement of the miRDB database allowed the identification of the four miRNA target transcripts (data not shown). Remarkably, a group of five genes was revealed to be potentially targeted by the three (out of four) intron-hosted (common) miRNAs. For example, *CAMK1D*, *PRR11*, and *SUSD6* (*KIAA0247*) could be simultaneously targeted by miR-1273g-3p, miR-619-5p, and miR-5096 molecules (Table 1). Besides the four aforementioned intron-accommodated miRNAs, additional species likely originated from each one of the three retained introns may critically contribute to the presumable repression of *HGF*, *TSTD3*, *PRR11*, *CAMK1D*, and *SUSD6* genes (Table S2).

To subsequently generate and visualize molecular interactomes of the proteins encoded by the five, herein identified, intronic miRNA target transcripts, we suitably employed the IntAct and Cytoscape bioinformatics subroutines. Except from TSTD3, all other four proteins led to protein–protein interaction maps of one member for PRR11 (Figure 4B), two members for SUSD6 (Figure 4C), eight members for CAMK1D (Figure 4D), and seven members for HGF (Figure 4E). Since c-MYC was unveiled as a protein partner of PRR11 (Figure 4B) and *c-MYC* gene was shown to undergo melanoma-specific intron retention (Figure 1), all five intronic miRNA target genes and some additional ones encoding selected protein interactors were, next, examined for intron retention incidents.

### 2.5. Intronic miRNA Target Gene-Specific Silencing and Aberrant Splicing in Melanoma

To reinforce the mechanistic value of the retained intron-derived miRNAs (Figure 4A and Table 1), the expression profiles of *PRR11* (implicated in cell cycle progression and epithelial to mesenchymal transition [57,58,59]) and *CAMK1D* (controls mitotic entry of endothelial cells, angiogenesis and epithelial to mesenchymal transition [60,61,62]) genes were investigated both in non-melanoma and melanoma specimens. In contrast to BCC and SCC (Figure S1A,C), melanoma (Figure S1B,D) biopsies proved to be characterized by transcriptional repression of *PRR11* (Figure S1A,B) and *CAMK1D* (Figure S1C,D) genes, as indicated by the amplification profiles of the expected 247 and 192 bp PCR products, respectively. Similarly, the other two intron-hosted miRNA target genes *HGF* (encodes for a growth factor that plays a causal role in cell survival, proliferation, angiogenesis, migration, metastasis, resistance to therapy, and epithelial to mesenchymal transition [63,64,65,66,67,68,69]) and *SUSD6* (a p53-inducible gene whose protein product regulates proliferation, angiogenesis and apoptosis [70,71,72]) were presented with undetected (*HGF*) (Figure S2A,B), or hardly detected (*SUSD6*) (Figure S2C,D), expression levels in melanoma (Figure S2B,D) but not in BCC and SCC (Figure S2A,C) patient cDNA collections, as dictated by the obtained amplification patterns of the expected 216 bp for *HGF* and 241 bp for *SUSD6* PCR fragments. *TSTD3* was presented with complete lack of gene expression in both non-melanoma and melanoma examined groups (data not shown). Intriguingly, *HGF* seemed to be subjected to aberrant splicing events, as evinced by the multiple and high molecular weight, diverse size PCR bands (asterisks) observed in BCC (Figure S2A) and melanoma (Figure S2B) specimens. A melanoma-specific similar mechanism of aberrant splicing seems to also operate for the *SUSD6* gene (Figure S2D; asterisks).

To further expand our study from intronic miRNA target genes to their protein product interactors, we, next, examined some—hitherto, not heavily analyzed—selected members of the two most multitudinous interactomes: CAMK1D (Figure 4D) and HGF (Figure 4E). The gene of CAMK1D interactor GMFG (a modulator of actin cytoskeleton organization, with a pivotal role in cell migration, invasion and epithelial to mesenchymal transition [73,74,75]), as indicated by the amplification profiles of the expected 237 bp long PCR fragment, was not suppressed in BCC and SCC (Figure S1E), or melanoma (Figure S1F) biopsy specimens, and, moreover, was not subjected to an irregular splicing process. On the other hand, the transcriptional activity of the gene that encodes the HGF interactor MEOX2 (GAX: a homeobox protein involved in the regulation of angiogenesis and resistance to chemotherapy [76,77,78,79]), as revealed by the obtained amplification patterns of the expected 147 bp long PCR band, could be detected—albeit at low levels—in BCC and SCC biopsy samples (Figure S1G), but was completely absent from melanoma ones (Figure S1H). Again, no indications of any *MEOX2*-specific aberrant splicing incidents could be recognized for all three types of skin cancer herein analyzed. Altogether, we suggest that a cluster of intronic miRNAs can successfully target a number of their cognate complementary transcripts, thus offering melanoma cells certain traits and/or advantages of the malignant phenotype. Some interactors of intron-hosted miRNA target gene protein products may remain unaffected, whereas other ones could undergo strong processes of gene repression and/or intron retention.

### 2.6. SRPX2 Gene Undergoes a Strong Intron Retention Process in Melanoma

**Figure 5:**
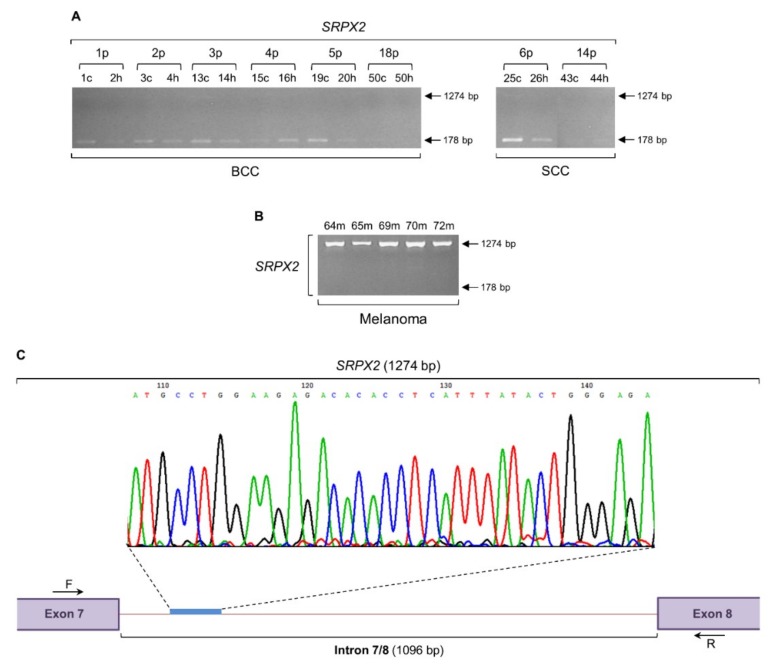
*SRPX2* gene is subjected to a strong intron retention mechanism in melanoma, but not in non-melanoma cells. Gene expression patterns of *SRPX2*, through engagement of RT-sqPCR protocols and utilization of total RNA extracts prepared from BCC and SCC (**A**) or melanoma (**B**) cDNA collections. *GAPDH* served as control gene (also, see Figure 6F and Table S1). (**C**) Representative DNA sequence chromatogram derived from the melanoma-specific 1274 bp long PCR product. A characteristic *SRPX2* intron 7/8 (1096 bp) sequenced area is indicated. Besides intron 7/8 retention (1274 bp), note the absence of *SRPX2* normal transcriptional activity (lack of 178 bp) in all melanoma biopsy samples (**B**). p: patient, c: cancer tissue (biopsy), h: healthy tissue (biopsy), m: melanoma (biopsy), bp: base pair, F: forward (primer) and R: reverse (primer).

The gene that encodes the HGF interactor SRPX2 (Figure 4E) proved to be subjected to a high intensity phenomenon of intron retention, specifically in melanoma samples (Figure 5). SRPX2 is a synaptogenic factor that critically contributes to the pathogenesis of language disorders [80,81,82], while it is also implicated in cell adhesion, migration and invasion [83,84,85], angiogenesis [86,87], resistance to therapy and epithelial to mesenchymal transition [88]. As indicated by the amplification profiles of the expected 178 bp long PCR product, in contrast to BCC and SCC (Figure 5A), melanoma samples proved to lack detectable levels of *SRPX2* regular gene expression (Figure 5B). However, a strong, high molecular weight PCR band of 1274 bp long was produced in all melanoma specimens examined (Figure 5B). Similarly to *Sestrin-1* (Figure 3A), a hardly detectable PCR fragment of 1274 bp could be also observed in the BCC and SCC patient cDNA collections (Figure 5A). Cycle sequencing of the purified, melanoma-specific PCR fragment of 1274 bp and subsequent alignment of the obtained DNA sequence with the reference one exported from the Ensembl Genome Browser 91 revealed the presence of a 1096 bp long DNA segment that represents the intron 7/8 (1096 bp) of *SRPX2* gene (ENST00000373004.4) (Figure 5C). Taken together, it seems that the absence of *SRPX2* legitimate transcriptional activity, together with the *SRPX2* strong intron retention process, can serve as a unified molecular signature for melanoma identification and its distinction from non-melanoma (BCC and SCC) skin cancers.

**Figure 6:**
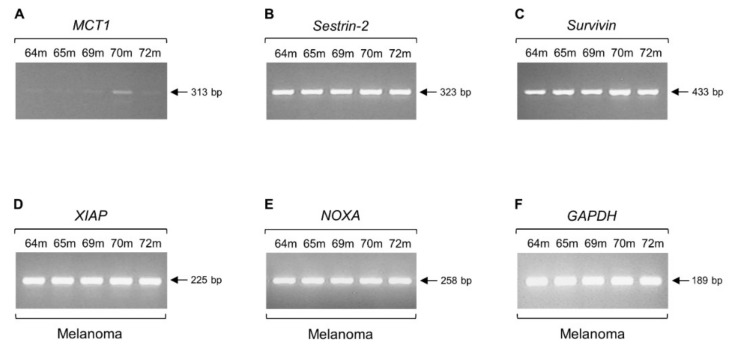
Lack of regular transcriptional activity is not a common mechanism in human melanoma. Expression profiles of a number of genes critically implicated in cell metabolism (**A**,**B**,**F**), cell signaling (**B**) and cell survival or death (**C**–**E**), via engagement of RT-sqPCR platforms based on total RNA extraction from melanoma biopsy specimens. (**A**) *MCT1* gene. (B) *Sestrin-2* gene. (C) *Survivin* gene. (**D**) *XIAP* gene. (**E**) *NOXA* gene. (**F**) *GAPDH* gene. Note the readily detectable (*MCT1*), or strongly activated (*Sestrin-2*, *Survivin*, *XIAP*, *NOXA*, and *GAPDH*) transcriptional expression of the herein examined genes. *GAPDH* was used as -housekeeping- gene of reference (also, see Figure 1, Figure 2 and Figure 3 and Figure 5, Table S1, and Figures S1 and S2). m: melanoma (biopsy) and bp: base pair.

To demonstrate that lack of regular transcriptional activity, such as the one observed in *c-MYC* (Figure 1B), *MCT4* (Figure 2B), *Sestrin-1* (Figure 3B), and *SRPX2* (Figure 5B) genes, does not represent a common phenomenon of melanoma cells, and to also validate the integrity of our melanoma RNA/cDNA preparations, next, we examined the expression profiles of a number of genes critically controlling cell metabolism, signaling, survival, and death (apoptosis). As illustrated in Figure 6, the *MCT1* (*SLC16A1*) (an MCT family member, regulator of lactate consumption, and *c-MYC* target [41,42,43,89]) (Figure 6A), *Sestrin-2* (*SESN2*) (a Sestrin family member, leucine sensor, and major regulator of mTOR signaling [48,49,50,90,91]) (Figure 6B), *Survivin* (*BIRC5*) (an inhibitor of apoptosis and modulator of resistance to therapy [92,93,94]) (Figure 6C), *XIAP* (*BIRC4*) (an inhibitor of apoptosis, with an important role in resistance to chemotherapy [93,95,96,97,98]) (Figure 6D), *NOXA* (*PMAIP1*) (a promoter of apoptosis and carrier of an ability to potentiate chemosensitivity [99,100,101]) (Figure 6E) and *GAPDH* (a key enzyme in glycolysis, iron metabolism, membrane trafficking, histone biosynthesis, DNA integrity maintenance, and receptor-mediated signaling [102]) (Figure 6F) genes, with *GAPDH* also serving as a housekeeping gene of reference (control) [103], were presented with either readily detectable (*MCT1*), or strongly upregulated (*Sestrin-2*, *Survivin*, *XIAP*, *NOXA*, and *GAPDH*) transcriptional activity patterns in melanoma biopsy specimens, directly reflecting the integrity, reliability and efficiency of transcription machineries in our melanoma biopsy cell populations. Notably, the absence of retained introns in the *MCT1* (313 bp), *XIAP* (225 bp), and *GAPDH* (189 bp) (and also *GMFG* (237 bp); Figure S1F) RT-sqPCR melanoma-specific products (Figure 6) (whose respective forward and reverse primers were designed to anneal within different exon sequences (Table S1)) serves as strong internal control/marker for the purity of our RNA/cDNA preparations, and demonstrates the RNA- and non-DNA-dependent origin of the retained introns in the *c-MYC*, *MCT4*, *Sestrin-1*, and *SRPX2* human melanoma RT-sqPCR fragments (Figure 1, Figure 2 and Figure 3 and Figure 5).

### 2.7. “ORFing” the c-MYC, MCT4, Sestrin-1, and SRPX2 Introns

Besides their presumable role as source of miRNA production (Figure 4), retained introns may cause premature termination of translation and/or synthesis of proteins encoded by intron-nested genes. Indeed, the presence of introns in all the herein examined genes *c-MYC* (2/3), *MCT4* (2/3), *Sestrin-1* (9/10), and *SRPX2* (7/8) was tightly associated with the in silico recognition of in-frame stop codons, either in each respective retained intron or its successive exon, thus likely resulting in the generation of truncated proteins with irregular or severely compromised activities (data not shown).

**Figure 7:**
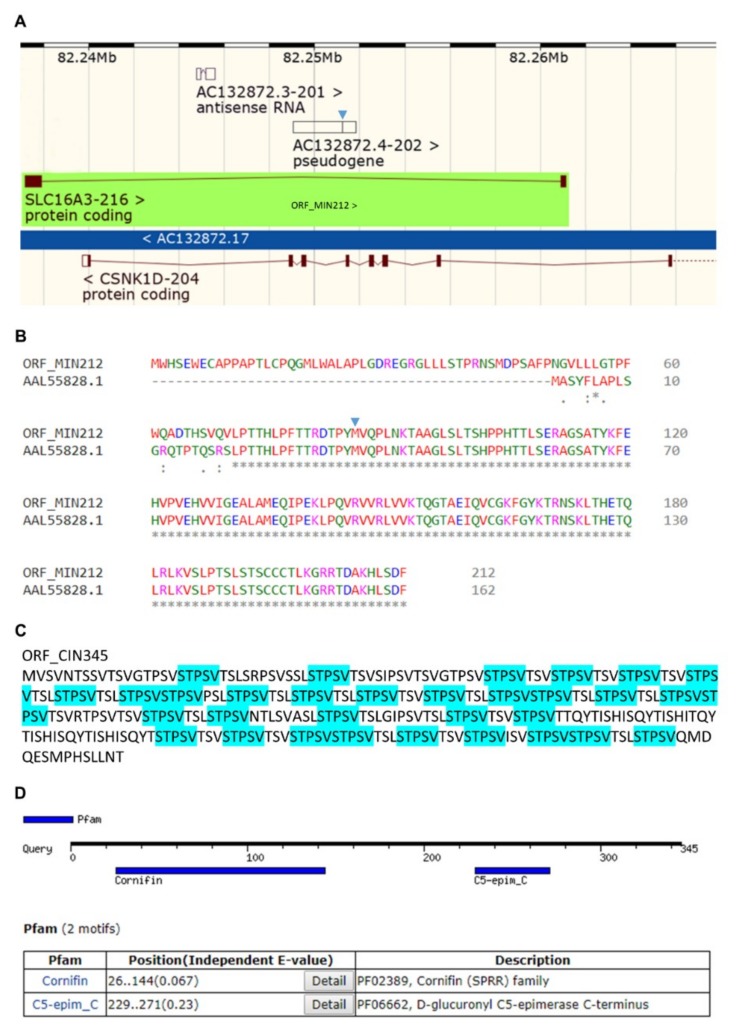
Identification of *MCT4/SLC16A3-216* intron-derived novel proteins: sequence homologies and functional domains. (**A**) Graphical presentation of the *MCT4/SLC16A3-216* splice-variant genomic area (modified from https://www.ensembl.org/Homo_sapiens/Gene/Summary?db=core;g=ENSG00000141526;r=17:82228397-82261129). Boxes denote the exons, while lines represent their in between introns. Note the DNA sequence overlap among *MCT4/SLC16A3-216* (splice variant), *AC132872.4-202* (pseudogene), and *HCKID/CSNK1D-204* (splice variant) gene territories. “>”: forward strand transcription, “<”: reverse strand transcription, arrowhead: pseudogene intron (“intron-in-an-intron”). Due to its large size, the *CSNK1D-204* transcript cannot be topologically aligned to the *SLC16A3-216* (and the *AC132872.4-202* pseudogene) one. (**B**) Amino acid sequence alignment of ORF_MIN212 to AAL55828.1 protein, via employment of Clustal Omega bioinformatics tool. The arrowhead indicates the amino terminal end of the second exon ORF that belongs to the *AC132872.4-202* pseudogene. Note that retention of the intron (**A**) expands ORF length and its similarity to AAL55828.1 further upstream towards its amino terminal direction. (**C**) Amino acid sequence of the ORF_CIN345 protein, as revealed by engagement of the ORF Finder bioinformatics platform. Fonts with blue shading indicate the “STPSV” repeats. (**D**) Detection of functional domains, in the form of “Cornifin” (26–144) and “C5-epim_C” (229–271) protein sequence motifs, in the ORF_CIN345 protein, through employment of the MOTIF Search bioinformatics tool.

Providing that not just the one identified in the present study, but all the introns of each gene transcript (*c-MYC*, *MCT4*, *Sestrin-1*, and *SRPX2*) can be potentially retained in human melanoma, next, we attempted to map their intronic landscapes for protein encoding sequences. Hence, by appropriately engaging the ORF Finder bioinformatics platform, with a minimal ORF length set at 300 nucleotides (and for certain cases, such as the *Sestrin-1* gene, 75 nucleotides), we were able to identify, for the first time, a number of interesting ORFs within selected introns of the examined genes. An ORF of 212 amino acid residues was recognized within the big intron 1/2 (23005 bp) of *MCT4/SLC16A3-216* (ENST00000583025.1) transcript variant and, surprisingly, it proved to derive from the intron-nested pseudogene *AC132872.4-202* (ENST00000622924.2), providing that it could also retain its internal intron (Figure 7A, arrowhead). In other words, the double intron retention (“intron-in-an-intron”) could allow the expression of ORF_MIN212 (MIN: MCT4 INtron; 212: number of amino acids) novel putative protein. Interestingly, a BLAST-P-mediated ORF_MIN212 alignment against nonredundant protein sequences (nr) unveiled its complete identity in 142 (71-212) amino acids with a human protein of 162 (21–162) amino acids (GenBank: AAL55828.1), predicted to be translated by a cDNA clone sequence of 2787 bp (GenBank: AF318321.1) that was previously reported to carry functions related to inhibition of cancer cell growth (Zhou et al., 2000; unpublished data; https://www.ncbi.nlm.nih.gov/nuccore/18027733). Optimization of sequence alignment in between ORF_MIN212 and AAL55828.1 putative proteins was performed via the suitable employment of Clustal Omega bioinformatics tool (Figure 7B). Mechanistically, the respective ORF hosted within the second exon of *AC132872.4-202* pseudogene (Figure 7B, arrowhead; “MVQPL … HLSDF”) is further expanded towards its amino terminal end via retention of the tiny preceding intron 1/2 (“AGGTC”) (Figure 7A, arrowhead), ultimately leading to a significantly bigger protein with an even higher level of homology with the AAL55828.1 protein (Figure 7B).

A second large ORF of 345 amino acids was recognized within the opposite (anti-sense) strand of *MCT4/SLC16A3-216* intron 1/2 gene sequence. Given that the *MCT4/SLC16A3-216* and *HCKID/CSNK1D-204* (ENST00000398519.9) gene sequences significantly overlap to each other and are transcribed from opposite DNA strands (https://www.ensembl.org/Homo_sapiens), the ORF_MIN345 putative protein could most likely derive from retention of the *HCKID/CSNK1D-204* intron 2/3 (10108 bp) and as such should be renamed ORF_CIN345 (C: CSNK1D). Strikingly, ORF_CIN345 seemed to carry the amino acid sequence “STPSV” repeated 32 times throughout the whole protein length, usually followed by variations of the “TSV(L)” tripeptide (Figure 7C). Furthermore, via employment of the MOTIF Search tool, two distinct motif sequences could be recognized within the ORF_CIN345 putative protein; the “Cornifin (SPRR)” (26–144 amino acids) and “C5-epim_C (D-glucuronyl C5-epimerase C-terminus)” (229–271 amino acids) ones (Figure 7D). SPRRs are small proline rich proteins that critically contribute to the production of cornified envelopes, which represent cross-linked matrices surrounding differentiated keratinocytes [104]. The property of SPRR3 family member to belong to a molecular signature that distinguishes metastatic from primary melanoma [105] strongly suggests for a critical role of ORF_CIN345 and, therefore, its respective retained intron, in the process of melanomagenesis.

**Figure 8:**
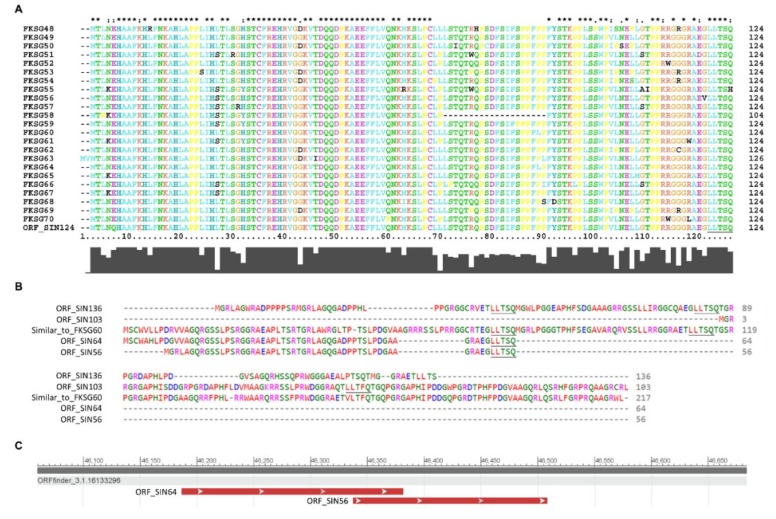
*Sestrin-1* intron 1/2 serves as a source for production of “LLTSQ”-containing proteins: recognition of novel FKSG and FKSG-like superfamily members. (**A**) Multiple protein alignment of the herein identified ORF_SIN124 to the FKSG (48–70) protein family members (categorized by name), via utilization of the Clustal X2 bioinformatics program. Note the striking similarities in amino acid sequence among all compared proteins. (**B**) Multiple protein alignment, via engagement of the Clustal Omega bioinformatics platform, of the herein predicted, to be produced, ORF_SIN136, ORF_SIN103, ORF_SIN64, and ORF_SIN56 proteins, to the similar-toFKSG60 previously reported one. Remarkably, note the presence of “LLTSQ” (or, its variant version “LLTFQ”) pentapeptide (indicated by underlined fonts) not only in all five of them (**B**), but also in the new FKSG (FKSG71) member ORF_SIN124 (**A**). (**C**) Graphical presentation of the ORF_SIN64 and ORF_SIN56 coding territories, as they result from ORF Finder bioinformatics application.

Since *c-MYC* and *SRPX2* genes were both presented with intronic ORFs of low or unknown predicted importance (data not shown), we, next, examined the coding landscapes of *Sestrin-1* introns. Among them, intron 1/2 (91451 bp) of *Sestrin-1/SESN1-204* (ENST00000436639.6) transcript variant proved to accommodate some of the most interesting ORFs. A group of five different ORFs were unveiled to contain at least once the “LLTS(F)Q” amino acid sequence. Remarkably, ORF_SIN124 (S: Sestrin-1) that represents a putative protein of 124 amino acids exhibited a striking similarity to all 23 (categorized by name) members of the FKSG (48-70) protein family (an initial approach via BLAST-P, successively optimized by Clustal X2 search) (Figure 8A). Furthermore, the other four intron-derived, putative proteins ORF_SIN136, ORF_SIN103, ORF_SIN64, and ORF_SIN56, with a number of 136, 103, 64, and 56 amino acids, respectively, shared significant similarities not only among themselves, but also to the Similar_to_FKSG60 (partial) human protein of 258 amino acids (BAD92674.1) (an initial approach via BLAST-P, successively optimized by Clustal Omega search) (Figure 8B). Intriguingly, although ORF_SIN64 and ORF_SIN56 represent distinct ORFs that derive from different codon frame utilization, they not only prominently overlap (in terms of topology) to each other, but also encode almost identical proteins (Figure 8C). Maybe the translation machinery in an intronic environment is prone to errors. If so, cells might engage different reading frames, in order to ensure the synthesis of particular proteins strongly implicated in the regulation of melanoma initiation and progression. To our knowledge, hitherto, no published report (in PubMed search engine) exists concerning the association of FKSGs with human cancer. Hence, providing that the *Sestrin-1* intron-derived FKSG and FKSG-like novel proteins are indeed produced, as herein predicted, a network of “LLTS(F)Q”-dependent interactions among FKSG and FKSG-like superfamily members may be activated to potentiate the malignant character of melanoma.

### 2.8. Molecular Modeling of “LLTSQ”- or “STPSV”-Rich Proteins

Given that, among its group homologous members, ORF_SIN136 is the only putative protein that carries two complete “LLTSQ”, one truncated (“LLTS”), and one variant (“LPTSQ”) motif, next, we employed BLAST-P to search for sequence alignments of ORF_SIN136 with proteins containing the “LLTSQ” pentapeptide. Among the top twenty most homologous proteins, the Low Quality Protein: Collagen_Alpha-1(I)_Chain-Like (*Nomascus leucogenys*) (for reasons of simplicity, herein abbreviated as CA1ICL-861) protein of 861 amino acids (NCBI Reference Sequence: XP_012359405.1), besides its strong similarity to ORF_SIN136, was presented to also carry the highest number of “LLTSQ” repeats (Figure S3A and data not shown). Indeed, in CA1ICL-861, the “LLTSQ” motif is repeated 19 times throughout the whole protein length, while the “LLTYQ” one can be identified two more times, with some additional motif variations (e.g., “LLTSH”, “LLTSL”, “LLISQ”, and “LLSSQ”) also being recognized in the protein (Figure S3A). Surprisingly, the “*AEA*LPTSQ*mMG*” (fonts in italics denote the amino acids surrounding the -variant- core motif) motif of CA1ICL-861 strikingly resembles the “*AEA*LPTSQ*tMG*” one observed in the ORF_SIN136 putative protein. Moreover, the distinct ORF_SIN81 putative protein that contains two “LLTSQ” motifs and shares with ORF_SIN136 the consensus sequences “AAGRRxSxLx(x)RxGxQAEGLLTSQT” and “QAExLLTSQTG(x)RPGR”, in a BLAST-P alignment search, also exhibited (among others) strong homologies with the CA1ICL-861 protein (data not shown).

Hence, given the presumable importance of the “LLTSQ” rich CA1ICL-861 protein, next, we attempted, via the engagement of I-TASSER online server, to predict its 3D structural model (Figure S3B). A similar bioinformatics protocol was also applied for the “AAGRRGSSLLIRGGCQAEGLLTSQT” extended motif of ORF_SIN136 (Figure S3C) and its highly similar “AAGRRRSSLSRLGSQAEGLLTSQT” respective one of ORF_SIN81 putative protein (Figure S3D). To the same direction, and investigating the other pentapeptide rich ORF_CIN345 protein that carries the “STPSV” motif 32 times, a molecular model of the protein was additionally produced (Figure S3E). In order to estimate the quality of the predicted models by I-TASSER, a confidence score was calculated, the C-score, which typically ranges from −5 to +2. A high value of C-score indicates high confidence in the model. Model predictions were evaluated using the template modeling-score (TM-score) and root mean-square difference (RMSD). TM-score is a scale for measuring the structural similarity between two proteins with different tertiary structures. A TM-score > +0.5 indicates a model with correct topology, while a TM-score < +0.17 indicates random similarity. The values of C-score, TM-score, and RMSD for the three predicted models are presented in Figure S3F. These values indicate that all molecular models, but especially the one concerning the CA1ICL-861 tertiary structure (Figure S3B), are reliable and of high quality. The ability of ORF_SIN136 and ORF_SIN81 motifs to acquire specific and distinct structural conformations (Figure S3C,D) further strengthens their potential proficiency to serve as new functional “microdomains” in protein physiology (e.g., mediating/regulating protein–protein interactions). The ability of CA1ICL-861 to obtain such a nicely organized molecular structure presumably reflects, among others, the structural value of “LLTSQ” repeats, and as such, together with the other novel “LLTSQ”-containing intronic ORFs, unveils the functional significance of intron retention and intron-derived ORFs in human melanoma. Interestingly, both CA1ICL-861 and ORF_CIN345 proteins seem to be tertiary-structured in a “horseshoe” shape, with a molecular symmetry axis dividing each protein into two distinct parts. If so, a mechanism of pentapeptide repeat-dependent protein–protein interactions that may cause formation of polymer-based networks is herein proposed to operate in melanoma.

### 2.9. The “LLTSQ”-Rich Protein CA1ICL-861 Is Predicted to form Dimers and Tetramers, and to Also Recognize Acetylated Lysine

**Figure 9:**
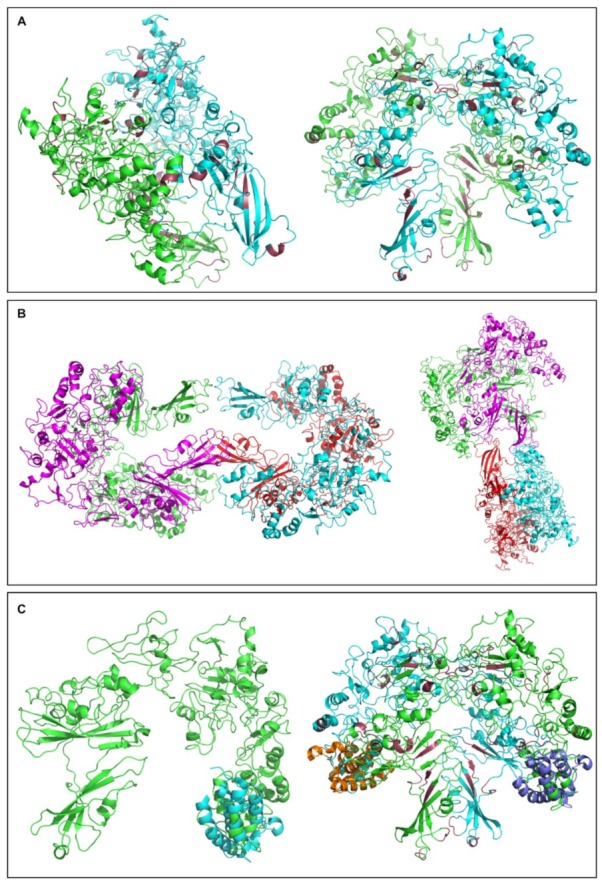
CA1ICL-861 can in silico generate dimers and tetramers, and also obtain a bromodomain-like structure. (**A**) Molecular model (best views), via HADDOCK2.2 engagement, of the predicted CA1ICL-861 homodimer. The “H”-like (left panel) and “tree”-like (right panel) structures are illustrated. Each monomer is presented with different color. The raspberry color indicates the “LLTS” tetrapeptide repeats. Note the molecular symmetry of the complex. (**B**) Molecular model (best views), through HADDOCK2.2 employment, of the in silico structured CA1ICL-861 homotetramer. Each monomer is pictured with different color. Note the remarkable molecular symmetry of the complex (left panel). (**C**) Structural alignment (best views), by PyMol “Align Command” utilization, of the CA1ICL-861 monomer (left panel) or dimer (right panel) with chain A of human CBP bromodomain (experimentally determined structure; PDB ID: 4OUF). CBP bromodomain is illustrated with light blue (left panel: CA1ICL-861 monomer) or a slate and orange (right panel: CA1ICL-861 dimer) color. Each monomer is shown with different color (right panel). The raspberry color indicates the “LLTS” tetrapeptide repeats (right panel). Note the partial, but significant, superposition of CBP bromodomain on CA1ICL-861 modeled structure. Also, observe the 2:2 molecular stoichiometry between the structurally aligned CA1ICL-861 dimer and CBP bromodomain protein models (right panel).

Driven docking experiments were performed using as structural platform the tertiary molecular model of CA1ICL-861 protein (Figure S3B), in an effort to predict if it can produce homodimers or homotetramers. To accomplish this, the HADDOCK2.2 web server was appropriately engaged. Our first attempt regarded the possible formation of homodimers. Since there was no previous evidence in terms of protein–protein interface residues, the CPORT consensus algorithm was used. The best solution obtained exhibited a HADDOCK score of −232.3, with the values of energies (Kcal/mole) estimated as follows; (a) van der Waals energy: −173.1; (b) electrostatic energy: −329.7; (c) desolvation energy: −39.8; (d) restraints violation energy: 464.7; and (e) total buried surface area (BSA): 5891.1 Å^2^. The high value of restraints violation energy has derived from system’s “peculiarity” that many of protein–protein interface residues predicted by the CPORT algorithm were not taken into account during the docking process. Two characteristic views of the best solution regarding the constructed dimer are illustrated in Figure 9A (left and right panels). An “H”-like (left) and “tree”-like (right) molecular shape can be recognized. Given the in silico ability of CA1ICL-861 protein to form homodimers, next, we examined its structural proficiency to produce tetramers. A similar strategy was applied, and the best solution for CA1ICL-861 homotetramer was presented with a HADDOCK score of −97.4 and energy values (Kcal/mole) as follows; (a) van der Waals energy: −52.8; (b) electrostatic energy: −186.4; (c) desolvation energy: −22.4; (d) restraints violation energy: 149.8; and (e) total buried surface area (BSA): 2013.6 Å^2^. Two representative views of the best solution that correspond to the homotetramer structure of CA1ICL-861 protein are shown in Figure 9B (left and right panels). It must be a specific “LLTSQ”-dependent conformation that propels CA1ICL-861 to generate dimers and tetramers. Providing their multimerization, filament-like structures may arise under certain cellular settings. Similarly, our intron-derived ORFs that carry the “LLTSQ” motif could be also assembled in filamentous-like networks (presumably containing the FKSG/FKSG-like superfamily members) exclusively in melanoma environments.

Since the BLAST-P-mediated homology search of ORF_SIN103 and Similar_to_FKSG60 proteins unearthed their association with the bromodomain superfamily (data not shown), and given the comparatively low fidelity and confidence values of their respective molecular models (data not shown), next, we examined if the “LLTSQ” rich CA1ICL-861 protein also carries a bromodomain-like structure. Hence, to identify if part of the CA1ICL-861 molecular model shares some structural similarity to a typical bromodomain, structural alignment was performed by suitably employing the “Align Command” of PyMol Molecular Visualization System. “Align Command” performs a sequence alignment followed by a structural superposition and then carries out zero or more cycles of refinement in order to reject structural outliers detected during the fit. In our case, we used the in silico model of CA1ICL-861 monomer (Figure S3B) or dimer (Figure 9A), and chain A of the experimentally determined structure of human CBP bromodomain [106] (PDB ID: 4OUF). The resulted RMSD value was estimated at 4.322 Å, while 19 Ca atoms were successfully aligned. As illustrated in Figure 9C, part of the CBP bromodomain can be nicely superposed on both CA1ICL-861 monomer (left panel; 1:1 stoichiometry) and dimer (right panel; 2:2 stoichiometry) modeled structures. Since bromodomains are able to specifically interact with acetylated lysine residues [107,108], an acetylated lysine-dependent interactome of CA1ICL-861 may critically operate under certain physiological or oncogenic conditions. It must be the “LLTSQ” pentapeptide repeats that strongly support the tertiary conformation of bromodomain-like structure (Figure 9A,C; raspberry color). If so, any “LLTSQ”-mediated protein–protein interaction among our intron-hosted ORFs, FKSG/FKSG-like members, and CA1ICL-861 (human counterpart) protein could decisively modulate bromodomain-like activities to recognize acetylated lysines in a number of target proteins, including the ones associated with chromatin, thus essentially regulating gene transcription in melanoma, but not in non-melanoma cell contexts.

Altogether, it seems that (a) the strong sequence homologies observed in between our intron-derived novel ORFs and previously reported proteins, (b) the remarkably high number of pentapeptide repeats in some of them or their close homologs, and (c) the capacity of selected superfamily members to produce 3D structural models, with presumable oncogenic functions, strongly suggest a critical role of retained introns as sources in synthesis of new proteins with likely cardinal contribution to the regulation of skin cancer development and response to therapy.

## 3. Discussion

Tumor heterogeneity, which drives tumor evolution and metastasis, is a key challenge in cancer biology and medicine. As a result of heterogeneity, a tumor may contain a diverse collection of cells carrying distinct molecular signatures with different levels of sensitivity to therapy. Preexistence of subclonal populations or evolution of drug-tolerant cells can frequently emerge as selective responses of a tumor to the therapeutic pressure applied [109,110]. Since all types of cutaneous malignancies can develop resistance to drug treatment [67,68,69,111,112,113,114,115], novel oncogenic processes and regulators are necessitated to be efficiently targeted to eliminate therapy refractory tumors. Notably, it seems that alterations in the RNA splicing machinery and its components may cause novel oncogenic addictions and/or vulnerabilities that can be therapeutically exploited, using compounds targeting either the irregular splicing process or its aberrant products [26]. Remarkably, an orally administered, small-molecule, splicing modulator (H3B-8800) has recently proved to potently and preferentially kill spliceosome mutant tumor cells due to their property to retain certain GC-rich, short, introns [116]. Therefore, melanoma tumor subpopulations carrying intron retention events could be efficiently targeted by such type of novel drugs.

The seminal discovery of RNA spliceosomal mutations to likely act as cancer drivers [26] has underscored the need to identify critical mechanistic connections between noncanonical RNA splicing and tumorigenesis. Characteristic is the example of an aberrantly spliced (lacks exons 4–8) *BRAF^V600E^* transcript that encodes a variant protein providing dimerization-mediated resistance of melanoma cells to the drug Vemurafenib [67,115]. On the other hand, the mechanistic relationship between *CASP8* transcript variants, carrying retained sequences from intron 8, and BCC risk remains tentative and merits further exploration [10]. However, intron retention has emerged as a widespread mechanism for the inactivation of tumor suppression regulators (e.g., CASP8). The higher frequency of intron retention for tumor suppressor knockdown in comparison to exon skipping has been previously associated with the percentage of premature termination codons (PTCs) generated. It seems that in contrast to ~97% of the observed intron retention incidents, only ~50% of the exon skipping events can produce PTCs. Furthermore, disrupted reading frames may be more prone to cause loss of function in tumor suppressors than gain of function in oncogenes [32].

Providing that intron retention may serve more than a “simple” PTC source, not only tumor suppressors but also oncogenes and other type of genes as well could be similarly subjected to this process. In accordance, *c-MYC* gene was herein shown to undergo intron retention (Figure 1), thus indicating novel functions of its nonspliced intron(s) in human melanomagenesis. It has been previously reported that c-MYC maintains the splicing fidelity of exons carrying a weak 5′ donor site, while perturbation of its signaling axis can result in either intron retention or exon skipping events [34]. Therefore, the absence of *c-MYC* regular transcriptional activity in melanoma, but not in BCC and SCC biopsies (Figure 1), may render cells vulnerable to an intron retention process. Only selected transcripts are subjected to this type of irregular splicing, with *c-MYC* likely representing one of the key targets, in a “positive feedback loop” operating in a way that the lack of *c-MYC* canonical transcript fosters *c-MYC* intron retention, which in turn, due to the generated PTCs, ensures in a second level the absence of regular c-MYC protein production.

This c-MYC-dependent triggering of intron retention presumably compels the *Sestrin-1* and *SRPX2* genes to undergo a similar process of noncanonical splicing (Figure 3 and Figure 5), likely providing melanoma cells with certain survival, growth, and motility advantages. The absence of *c-MYC*, *Sestrin-1*, and *SRPX2* regular transcriptional activity, together with the retention of specific introns in their respective transcripts (Figure 1, Figure 3 and Figure 5), seem to comprise a novel, reliable and powerful composite molecular signature for the genotypic distinction of human melanoma from BCC and SCC non-melanoma tumors. Although our cohorts do not include large number of patients, all biopsy specimens per cohort are presented with unvarying expression profiles of the respective introns being retained in the three herein examined genes, thus demonstrating the significance of gene-specific, strong intron retention as a diagnostic biomarker for melanoma. Since the intron retention pattern of *MCT4* gene is observed both in melanoma and non-melanoma samples (Figure 2), it cannot by itself serve as a mechanistic biomarker for melanoma. Nevertheless, the lack of its regular transcriptional activity, combined with the strong retention of intron 2/3, could likely enrich our three-gene molecular signature with one additional member.

If so, a mechanistic explanation has to be given for the *MCT4* gene expression profile of BCC patient 14h (3p) that has proved almost identical with the one of melanoma patients (Figure 2). To answer this, we suggest that the healthy tissue of BCC patient 14h (3p) has lost its differentiation features and has become premalignant, thus endowing the intron retention and lack of canonical transcription of *MCT4* gene with a prognostic power for skin cancer development. Alternatively, given that all and not just the one herein examined (2/3) introns of *MCT4* are strongly retained, distinct transcripts derived from either the forward or the reverse strand of the same genetic area may be differentially produced and differentially affect the healthy versus the cancer tissue. Strikingly, the *MCT4/SLC16A3-216* intron 1/2 accommodates the *AC132872.4-202* nested pseudogene and notably overlaps with the *CSNK1D-204* gene sequence, with *SLC16A3-216* being transcribed from the forward and *CSNK1D-204* from the reverse strand (Figure 7). If the *SLC16A3-216* intron 1/2 (*AC132872.4-202*)-hosted ORF_MIN212 is selectively synthesized in healthy cells, and given the potential tumor suppressor function of its highly homologous protein AAL55828.1 (Zhou et al., 2000; unpublished data) (Figure 7), a role could be given in ORF_MIN212 to inhibiting skin cancer. In contrast, the *CSNK1D-204* intron 2/3-derived ORF_CIN345 could be exclusively produced in melanoma (and maybe BCC or SCC), but not in surrounding stromal (healthy) cells, in order to maintain their malignant phenotype, or promote subsequent metastasis. In accordance, the presence of a “Cornifin” (or “Cornifin”-like) motif, usually recognized in the SPRR family members [104], and their ability to critically control tumorigenesis [104], dictates the major contribution of ORF_CIN345 to melanoma. Since *SPRR3* belongs to a gene signature that can discriminate primary from metastatic melanoma [105], we herein suggest that ORF_CIN345 may promote melanomagenesis via perturbation of the SPRR-dependent cornification process. A “decornification” event may be required for metastasis to occur in human melanoma.

The *MCT4* intron retention in both melanoma and non-melanoma tumors (Figure 2), besides its mechanistic value, also provides us with technical guarantees concerning the efficiency and fidelity of the RT-sqPCR protocols herein applied. It seems that the absence of random primers (6-mers) from the RT reaction of BCC and SCC biopsy specimens does not harm our proficiency to detect retained intron incidents, while their (6-mers) presence in the respective protocols for melanoma samples may not cause beneficial results, since the short distance in between the annealed random 6-mers could not allow the generation of long transcripts, like the ones identified for our *c-MYC*, *Sestrin-1*, and *SRPX2* genes undergoing melanoma-specific intron retention. It must be the physical distance from poly(A) (A: adenine) tail (the annealing site of oligo-dT primer) that controls the detection efficiency of an intron retention event; the longer the distance the lower the efficiency. This and the limited capacity of MMLV to reverse transcribe templates longer than 5–7 Kb (depending on the sequence complexity) maybe some of the reasons that the observed intron retention frequency could be significantly reduced as compared to its actual in vivo presence and activity. Despite the utilization of new generation reverse transciptases, with stronger elongation power (e.g., 12 Kb), it remains still elusive to successfully examine noncanonical splicing and retention incidents of introns located close to the 5΄-end of big-sized genes.

Since the retention of respective introns in the three examined genes *c-MYC* (2/3), *Sestrin-1* (9/10), and *SRPX2* (7/8) caused the in silico disruption of their reading frames, due to introduction of intronic PTCs (data not shown), an NMD-mediated process would be expected to degrade these PTC-containing aberrant transcripts. NMD is an mRNA surveillance mechanism that recognizes and eliminates transcripts carrying PTCs, immediately after their entry into the cytoplasm [117,118,119]. However, the strong expression levels of *c-MYC* (1564 bp) (Figure 1), *Sestrin-1* (1253 bp) (Figure 3), and *SRPX2* (1274 bp) (Figure 5) irregular transcripts indicate their ability to escape the NMD quality control system operating in melanoma cells. In contrast, the hardly detectable PCR bands of intron-containing transcripts in BCC and SCC biopsy collections could be attributed, besides the compromise of intron retention process itself, to the generally unimpaired NMD activity in non-melanoma environments. On the other hand, melanoma cells may be able to adjust NMD machinery functions to allow survival of selected transcripts with PTCs. Transcript-specific resistance to NMD could represent a common feature of cancer transcriptomes. Confinement of certain transcripts retaining selected introns to the cell nucleus is one mechanism to overcome NMD-mediated degradation [120]. Similarly to neuronal activation responses [121], upon oncogenic stimulation, these, stored in the nucleus, irregular transcripts could undergo rapid intron excision and export to the cytoplasm, thus generating readily available RNA pools for protein synthesis. Alternatively, a global perturbation of NMD pathway in melanoma, but not in BCC or SCC disease, could not be overlooked. Given that the *UPF1* gene, whose product represents the core component of NMD machinery, is commonly mutated in pancreatic adenosquamous carcinoma [122], melanoma-specific NMD target transcripts with retained introns may be likely upregulated in a defective UPF1-dependent manner, providing cells with critical malignancy advantages, such as therapy resistance and metastasis.

Besides their roles in the production of truncated proteins (due to PTC generation) and nuclear compartmentalization of nonspliced transcripts (to evade NMD-mediated elimination), retained introns may critically contribute to melanomagenesis via distinct mechanisms of intron-derived ORFs. Under oncogenic conditions of intron retention, ribosomes may choose to specifically translate intronic ORFs—partly disregarding exon-based canonical synthesis—at least in a certain subpopulation of irregular transcripts. Apparently, intron-derived ORFs could be produced independently of an intron retention process. In addition to a presumable “decornification” activity of ORF_CIN345 (Figure 7), ORF_SIN124, and the other intronic proteins containing the “LLTSQ” sequence (ORF_SIN136, ORF_SIN103, ORF_SIN64, and ORF_SIN56) (Figure 8) could decisively regulate melanoma progression and metastasis. Strikingly, ORF_SIN124 proved to belong to the human FKSG family and to represent its 24th new member. *FKSG49* has been previously associated with early acute renal allograft rejection [123], while some expression profile data sets (gene chips) in skin cancer, including melanoma (https://www.ncbi.nlm.nih.gov/geoprofiles/?term=FKSG49+AND+melanoma), do not favor its prominent contribution to the disease. However, the remarkably strong homologies detected among all FKSG family members (Figure 8) argue against the specificity and reliability of the obtained *FKSG49* transcriptional patterns.

Hence, to somehow predict the molecular functions of ORF_SIN124 and its homologous ORF_SIN136, ORF_SIN103, ORF_SIN64, and ORF_SIN56 intronic proteins (Figure 8), tertiary structures were bioinformatically modeled, but they proved to lack high quality and confidence values (as compared to the CA1ICL-861 ones; see below) (data not shown). Next, as the best alternative, the “LLTSQ” rich CA1ICL-861 protein, and the “LLTSQ”-carrying motifs of ORF_SIN136 and ORF_SIN81 intronic proteins were also molecularly structured. A similar rationale and technical approach were applied for the ORF_CIN345 protein, as well. In contrast to ORF_CIN345, a highly organized molecular structure of alpha helices and beta strands was unveiled for CA1ICL-861 (Figure S3). Since ORF_CIN345 contains 32 times the “STPSV” repeat, it may be the proline (“P”) amino acid residue that breaks protein’s secondary structures. To examine this, we in silico remodeled a modified version of the protein in which a glycine (“G”) has replaced every proline in each repeat (“STGSV”). Interestingly, a number of alpha helices and beta strands are formed, with the general structure obtaining a “spring”-like shape (Figure S4). It is probably the prolines that provide the presumable “cornification” activity of ORF_CIN345, offering the required mechanical strength and elasticity for metastasis.

The ability of “LLTSQ”-carrying motifs in ORF_SIN136 and ORF_SIN81 proteins to acquire either a beta strand or an alpha helix secondary structure (Figure S3) likely indicates the structural flexibility of the sequence and its capacity to serve as a residue context-dependent “chameleon domain”. It could be the large number of “LLTSQ” repeats in CA1ICL-861 stabilize the protein and provide such a nicely organized structure (Figure S3). One or two repeats, like the ones observed in FKSG or FKSG-like superfamily members, may not suffice for a firm tertiary structure, thus suggesting the requirement of protein–protein multiplex interactions able to produce tight conformations in a solid network. In terms of structural dynamics, the striking proficiency of “LLTSQ” rich CA1ICL-861 protein to generate dimers and tetramers with high levels of intra- and intermolecular symmetry (Figure 9) strongly indicates the ability of intron-hosted “LLTSQ”-containing proteins to form filament-like structures that likely belong to oncogenic networks controlling metastasis and drug resistance.

The surprising structural resemblance of CA1ICL-861 to part of CBP (major transcriptional coactivator) bromodomain (Figure 9), which specifically recognizes acetylated lysine residues, such as the ones residing in the amino terminal tails of histones [106,107,108], likely unveils a novel molecular function of the protein. We suggest that at the level of CA1ICL-861 (human counterpart) dimer or tetramer, the presumable bromodomain-like structure obtains a better organized molecular shape to more efficiently recognize acetylated lysine residues on the chromatin proteins of melanoma cells, thus critically modulating acetylated lysine loads and transcriptional activities of genes controlling drug resistance and metastasis. “LLTSQ”-based presumable multimerization of our intron-derived ORFs and/or FKSG/FKSG-like superfamily members, and their putative protein–protein interactions with human CA1ICL-861 could result in perturbations of bromodomain-like activities that may operate in a chromosome-dependent manner, as possibly dictated by the identical number (24) of human chromosomes (22 autosomal and two sex) and FKSG (48–70 and ORF_SIN124) family members (Figure 8). Alternatively, intron-hosted ORFs and FKSGs could be assembled in dimers, tetramers or multimers that might serve as building units for bromodomain-like molecular constructions. Following previous strategies to block the classical CBP bromodomain [124,125], successful targeting of the, herein predicted, “LLTSQ”-dependent bromodomain-like structure(s) may prove therapeutically beneficial for the disease.

Besides intronic ORFs, intron-hosted miRNAs must also serve as a powerful mechanism to control melanomagenesis. Notably, in a previous report, the miR-619-5p, miR-5095, miR-5096, and miR-5585-3p species, all herein identified to presumably derive from sequences belonging to retained introns (Figure 4), were presented as a unique set of miRNAs that have hundreds of human target genes and also bind to their cognate transcripts with high affinities [126]. A large collection of miR-1273 family (whose member miR-1273g-3p was also herein recognized) binding sites on mRNA targets was also described by a different study [127]. The untranslated (5′ and 3′) region and coding domain sequences are all targets for the aforementioned miRNA species [126,127]. It seems that numerous genes can encode intronic miRNAs and these are transcribed in parallel with their host transcripts, likely requiring a slightly different mechanism of biogenesis as compared to the one of exonic miRNAs [128,129]. Interestingly, miR-5585-3p, miR1273g-3p, miR-5096, and miR-5095 have been previously characterized as intronic miRNA species critically implicated in gastrointestinal and breast cancer [129]. Furthermore, an essential role of miR-5096 (our top in value miRNA, in terms of target gene and score numbers; Table 1) in the invasion of glioblastoma cells has been recently described [130]. Given the plethora of their target genes, intron-accommodated miRNAs must regulate melanoma development through a multiple gene, multifaceted, and complicated manner. For example, the miRNA-mediated transcriptional repression of *PRR11* and *CAMK1D* oncogenic drivers in melanoma biopsies (Figure S1) could be mechanistically compensated by the production of *HGF* aberrant transcripts (Figure S2), whose products may provide malignant cells with strong survival and growth advantages, and also confer resistance to selected therapeutic agents, such as Vemurafenib or its structural analog(s) [67,68,69].

Since intron retention is able to regulate critical differentiation programs, such as the ones involved in granulocyte, megakaryocyte, and erythrocyte cell lineages [131,132], and to also maintain homeostasis of essential metabolites, such as O-linked β-N-acetylglucosamine (O-GlcNAc) and S-adenosylmethionine (SAM) [133,134], there must be an active mechanism that tightly controls its cellular dependency, gene/transcript specificity, and incident frequency. In accordance, inhibition of DNA methylation increases intron retention, while depletion of MeCP2, the methyl-CpG-binding protein 2 [135], in cells and tissues also enhances the process [136]. A similar inverse correlation between methylation level of an intron and its retention in cognate RNA transcripts has been recently observed in breast cancer [137]. Hence, a melanoma-specific demethylation mechanism that operates in a gene-dependent manner may compel the *c-MYC*, *Sestrin-1*, and *SRPX2* transcripts to retain their introns, strongly suggesting an epigenetic control of splicing integrity. Moreover, presumable demethylation of intronic sequences not only fosters their retention in NMD refractory transcripts, but also liberates their intrinsic capacity to produce specific miRNAs and ORFs that can critically promote melanomagenesis.

## 4. Materials and Methods

### 4.1. Study Population

The study included six BCC, two SCC, and five melanoma (one malignant nevus and four clear melanomas) biopsy specimens derived from hospitalized Greek patients who underwent therapeutic surgery. Adjacent normal matched tissues were also excised from BCC and SCC patient cohorts, following pathologist’s evaluation for absence of carcinogenic features. None of the patients received any type of neoadjuvant therapy prior to surgery for tumor removal. Our study was conducted according to the Declaration of Helsinki Ethical Principles (for medical research involving human subjects), as revised in the year 2008. The applied protocols were approved by the Institutional Review Boards of “Attikon” University Hospital, Athens, Greece (for BCC and SCC specimens) (“3719 on 27-02-2007”) and “Andreas Sygros” University Hospital, Athens, Greece (for melanoma specimens [138]) (“AΠ3000 on 16-05-2011”). Informed consent was given by all the participated patients. Patient and tumor characteristics are demonstrated in Table 2 (for BCC and SCC) and Table 3 (for melanoma).

### 4.2. Total RNA Extraction

Following pulverization of fresh-frozen BCC and SCC cancer and matched healthy-tissue (control) specimens, total RNA was extracted using the TRI Reagent^®^ (Molecular Research Center Inc., Ohio, USA), following manufacturer’s instructions. RNA pellet was dissolved in RNA Storage Solution (Ambion—Life Technologies—Thermo Fisher Scientific, Waltham, MA, USA) and appropriately stored at −80 °C, until further processing. Total RNA concentration was evaluated by absorbance measurement at 260 nm, in a BioSpec-nano UV–Vis Spectrophotometer (Shimadzu Corp., Kyoto, Japan). RNA structural integrity was visually confirmed by agarose gel electrophoresis. Archival melanoma samples [138], kept in RNA*later* RNA stabilization reagent (Qiagen, Redwood City, CA, USA), were released from the reagent, and approximately 20 mg of tissue per sample were disrupted and homogenized. Total RNA was extracted from each melanoma specimen using the RNeasy Mini Kit (Qiagen, Hilden, Germany), according to manufacturer’s instructions. Purified RNA preparations were stored at −80 °C in RNase-free ddH_2_O. RNA concentration was determined by measuring the absorbance at 260 nm in a SmartSpec™ Plus Spectrophotometer (Bio-Rad, Hercules, CA, USA).

### 4.3. RT-sqPCR

Regarding the BCC and SCC specimens, 1000 ng of total RNA were reverse transcribed in a 20 μL reaction, containing 40 U recombinant ribonuclease inhibitor, 5 μM oligo-dT primer, 500 μM dNTPs (mix), and 50 U MMLV reverse transcriptase (Invitrogen, California, USA), at 37 °C for 60 min. Heat inactivation of the enzyme was performed at 70° C for 15 min. In a similar manner, 1000 ng of total RNA derived from melanoma samples were reverse transcribed in a 20 μL reaction volume, using the PrimeScript™ RT Reagent Kit (Perfect Real Time) (Takara Bio, Shiga, Japan), following manufacturer’s protocol. The reaction mixture contained 5× PrimeScript Buffer (includes dNTPs and Mg^2+^), PrimeScript RT Enzyme Mix I (includes RNase Inhibitor), 50 pmol oligo-dT primer, and 100 pmol Random 6-mers. RT was performed at 37°C for 15 min, while reverse transcriptase was heat inactivated at 85 °C for 5 sec. BCC, SCC and melanoma cDNAs were amplified by sqPCR, with a Bio-Rad T100 Thermal Cycler (Bio-Rad), using gene-specific oligonucleotide primers (Table S1). Obtained PCR products were resolved in 2–3% agarose gels, according to standard procedures. *GAPDH* served as gene of reference (control).

### 4.4. DNA Sequencing of PCR Products

Cycle sequencing of purified PCR fragments was carried out with one of the PCR nucleotide primers, using the v3.1 BigDye Terminator Cycle Sequencing Kit (Applied Biosystems, Foster City, CA, USA). The Sephadex G50-purified cycle sequencing products were analyzed on an ABI Prism^®^ Genetic Analyzer. Obtained DNA sequences were aligned against reference human gene sequences from the Ensembl Genome Browser 91 (https://www.ensembl.org/index.html) (*MYC*/*c-MYC*: ENST00000621592.5; *SLC16A3*/*MCT4*: ENST00000581287.5; *SESN1*/*Sestrin-1*: ENST00000436639.6; *SRPX2*: ENST00000373004.4) and appropriately examined to identify regions of sequence homology.

### 4.5. Bioinformatics Analysis

#### 4.5.1. miRNA Alignments to Intron Sequences—miRNA Target Predictions

To identify miRNAs (microRNAs) that are aligned to the three intron sequences herein analyzed (*c-MYC*: intron 2/3; *MCT4*: intron 2/3; *Sestrin-1*: intron 9/10), we employed the miRbase database that represents an online available tool designed to search for homologs of miRNA sequences (http://www.mirbase.org) [139,140]. The online database miRDB was used for miRNA target prediction (http://www.mirdb.org) [141,142].

#### 4.5.2. Intronic Open Reading Frame (ORF) Identification

Potential protein segments encoded by intron sequences of the *c-MYC*, *MCT4*, *Sestrin-1*, and *SRPX2* genes were recognized via engagement of the web version of ORF Finder (ORFfinder) bioinformatics resource (https://www.ncbi.nlm.nih.gov/orffinder). Any query sequence larger than 50 Kb long was suitably subdivided into two fragments, while the minimal ORF length was set at 300 nucleotides, unless stated differently (e.g., 75 nucleotides).

#### 4.5.3. Protein Sequence Alignments

Conserved amino acid sequences were determined by the multiple sequence alignment (MSA) of Clustal X2 and Clustal Omega tools. Clustal Omega algorithm was provided by the EMBL-EBI bioinformatics web and programmatic tools framework (https://www.ebi.ac.uk/Tools/msa/clustalo). Clustal Omega uses seeded guide trees and Hidden Markov Models (HMMs) profile–profile techniques to generate high quality MSAs between three or more sequences [143,144]. BLAST-P (Protein BLAST) (https://blast.ncbi.nlm.nih.gov/Blast.cgi?PAGE=Proteins) algorithm was used to compare intron-derived putative proteins with an open source library of sequences, in order to identify statistically significant resemblances in between proteins above a certain threshold. The MOTIF Search (http://www.genome.jp/tools/motif) online -GenomeNet- bioinformatics tool was suitably employed for the identification of putative protein motifs in the intronic ORF sequences.

#### 4.5.4. Molecular Assembly of Protein Interactomes

To generate protein–protein interaction maps, we employed IntAct that provides a freely available, open source, database system and analysis tool for molecular interaction data being derived from literature curation or direct user submission (https://www.ebi.ac.uk/intact) [145]. Visualization of protein interactomes was achieved by Cytoscape that represents an open source software platform specifically designed for visualizing complex networks and integrating them with any type of attribute data (http://www.cytoscape.org) [146].

#### 4.5.5. Protein Molecular Modeling—Structural Prediction of Protein–Protein Interactions

Three dimensional (3D) predictions were generated by using I-TASSER (Iterative Threading ASSEmbly Refinement), an online server that is designed for automated protein structure and function prediction (https://zhanglab.ccmb.med.umich.edu/I-TASSER) [147,148], without changing the default parameters of the software. Structural models of protein sequences were constructed from multiple threading alignments and iterative structural assembly simulations. Comparison of the produced models with other known protein structures provides insights for the function of proteins being investigated [149]. Derived models were subjected to energy minimization by applying the “Minimize Structure” subroutine of the UCSF Chimera Software (https://www.cgl.ucsf.edu/chimera) [150]. Images containing structural models were prepared by the PyMol Molecular Visualization System (http://www.pymol.org). Predictions of protein–protein interactions (docking experiments) of selected structural models herein constructed were carried out via utilization of the “Prediction Interface” of HADDOCK2.2 web server [151,152]. In order to identify the protein–protein interface residues, the CPORT prediction algorithm was suitably employed [153]. HADDOCK score, being the weighted sum of intermolecular electrostatic (E_elec_), van der Waals (E_vdW_), desolvation (ΔG_solv_), and ambiguous interaction restraint (AIR) energies, was used to rank the generated poses. The resulted models were visualized with the PyMol Molecular Visualization System.

## 5. Conclusions

A new oncogenic signature of gene-specific intron retention that can distinguish melanoma from non-melanoma biopsy tissues has been herein unveiled. The collection of *c-MYC*, *Sestrin-1*, and *SRPX2* nonspliced transcripts clearly typify human melanoma and molecularly differentiate it from BCC and SCC skin cancers. Besides the generation of PTCs, which result in translational perturbations, intron-hosted miRNAs, and ORFs may also significantly contribute to the malignant character of melanoma cells. Providing the resistance of intron-carrying irregular transcripts to NMD surveillance mechanisms, a number of novel proteins are presumed to derive from retained introns, critically contributing to drug resistance and metastasis of the disease. Targeting the intron retention process itself and/or intron-emanated products may prove therapeutically beneficial for suffering patients. A transcriptome-wide profiling of intron retention and its association with therapy responses and malignancy grades will not only expand our mechanistic view of melanoma development, but will also offer new, powerful, and efficient tools, in the form of mechanism-driven biomarkers, for the successful clinical management of melanoma. Large-scale mapping of intronic “miRome” and “ORFome” landscapes in melanoma versus healthy cohorts will most likely deliver important prognostic and diagnostic information for further risk stratification of prone to, or affected by, melanoma individuals beyond our current clinical and molecular standards. Drugging the melanoma-specific intronic “ORFome” may open a new therapeutic window for the disease.

Altogether, our intron retention-based molecular signature may not only serve as an alternative diagnostic tool for rare cases of melanoma of uncertain origin, but most importantly may provide the clinic with a therapeutic target in order to eradicate intron retention-carrying cancer cell subpopulations via the utilization of H3B-8800-like drugs.

## Figures and Tables

**Table 1:** Collection of miRNA species that are likely produced by both *c-MYC* (2/3) and *Sestrin-1* (9/10) retained introns, and can simultaneously downregulate the same group of target transcripts. Name of human miRNA molecule, number of miRNA target genes (transcripts), name (symbol) of target gene, name (symbol) of gene undergoing intron retention, and target score (success level of miRNA complementary binding to target transcript sequence) are shown (also, see Figure 4 and Table S2). hsa: *Homo sapiens.*

miRNAs	Number of Genes	Target Genes	Intron Retained	Target Score
hsa-miR-5585-3p	2	*TSTD3*	*c-MYC* + *Sestrin-1*	50
		*HGF*		52
hsa-miR-1273g-3p	4	*PRR11*	*c-MYC* + *Sestrin-1*	50
		*SUSD6*		76
		*CAMK1D*		87
		*HGF*		89
hsa-miR-619-5p	4	*TSTD3*	*c-MYC* + *Sestrin-1*	58
		*PRR11*		65
		*SUSD6*		75
		*CAMK1D*		81
hsa-miR-5096	5	*TSTD3*	*c-MYC* + *Sestrin-1*	52
		*HGF*		71
		*PRR11*		74
		*SUSD6*		85
		*CAMK1D*		94

**Table 2:** Clinical and biological (tumor) characteristics of BCC and SCC Greek patients herein studied. Patient number, age ranges, lesion, affected tissue, ulceration, actinic (solar) elastosis, infiltration, and foci number are reported.

Patient Number	Age Ranges	Lesion	Affected Tissue	Ulceration	Actinic (Solar) Elastosis (Neighboring Papillary Chorion Tissue)	Infiltration	Foci Number
1	80–85	BCC	Nose (Left Pterygium)	Yes	Yes	No	2
2	70–75	BCC	Cheek	Yes	Yes	No	1
3	70–75	BCC	Eye (Right Upper Eyelid/Inner Canthus)	No	Yes	No	2
4	70–75	BCC	Eye (Left Inner Canthus)	No	Yes	No	1
5	80–85	BCC	Cheek (Right)	No	No	Yes (Subcutaneous Fat Tissue)	1
6	65–70	SCC	Forehead	No	Yes	Yes (Subcutaneous Fat Tissue)	1
14	85–90	SCC	Cheek (Left)	Yes	Yes	No	1
18	70–75	BCC	Dorsum (Back)/Thoracic Wall	Yes	No	Yes	Several (Some with BCC-SCC Features)

**Table 3:** Clinical and biological (tumor) characteristics of melanoma-affected Greek patients herein investigated. Patient number, age ranges, lesion, histogenic type, growth pattern, lymphocyte infiltration, mitotic index, ulceration, epidermal infiltration, neurotropism, regression, satellite foci, and Clark staging are shown.

Patient Number	Age Ranges	Lesion	Histogenic Type: Nodular Features	Growth Pattern	Lymphocyte Infiltration	Mitotic Index	Ulceration	Epidermal Infiltration	Neurotropism	Regression	Satellite Foci	Clark Staging: Melanoma Invasion
64	80–85	Malignant Nevus	No	Horizontal	Absent	0–5 mitoses/mm^3^	No	No	No	No	No	I–III: Early Stage
65	60–65	Melanoma	No	Horizontal	Absent	0–5 mitoses/mm^3^	No	No	No	No	No	I–III: Early Stage
69	80–85	Melanoma	No	Horizontal	Absent	0–5 mitoses/mm^3^	No	No	No	No	No	I–III: Early Stage
70	45–50	Melanoma	No	Horizontal	Brisk	0–5 mitoses/mm^3^	No	Yes	No	Yes	No	I–III: Early Stage
72	80–85	Melanoma	No	Horizontal	Brisk	0–5 mitoses/mm^3^	No	Yes	No	Yes	No	I–III: Early Stage

## Supplementary Materials

Supplementary materials can be found at https://www.mdpi.com/1422-0067/20/4/937/s1.

## Abbreviations

BCC	basal cell carcinoma
NMD	nonsense-mediated mRNA decay
PCR	polymerase chain reaction
RT	reverse transcription
SCC	squamous cell carcinoma
sq	semi-quantitative
UV	ultraviolet
