# Supplementary material for: Gene-Specific Intron Retention Serves as Molecular Signature that Distinguishes Melanoma from Non-Melanoma Cancer Cells in Greek Patients
Source: Int J Mol Sci. 2019 Feb 21;20(4):937. doi: 10.3390/ijms20040937 (PMC6412294; doi:10.3390/ijms20040937)
Supplement: Supplementary file 1 [file ijms-20-00937-s001.pdf]

Article-Supplementary File

Gene-Specific Intron Retention Serves as Molecular Signature that Distinguishes Melanoma from Non-Melanoma Cancer Cells in Greek Patients

Aikaterini F. Giannopoulou [1,†], Eumorphia G. Konstantakou [1,†,‡], Athanassios D. Velentzas [1,†], Socratis N. Avgeris [2], Margaritis Avgeris [3], Nikos C. Papandreou [1], Ilianna Zoi [4], Vicky Filippa [5], Stamatia Katarachia [1], Antonis D. Lampidonis [1], Anastasia Prombona [6], Popi Syntichaki [5], Christina Piperi [4], Efthimia K. Basdra [4], Vassiliki Iconomidou [1], Evangelia Papadavid [7], Ema Anastasiadou [5], Issidora S. Papassideri [1], Athanasios G. Papavassiliou [4], Gerassimos E. Voutsinas [2], Andreas Scorilas [3] and Dimitrios J. Stravopodis [1,*]

1 Section of Cell Biology and Biophysics, Department of Biology, School of Science, National and Kapodistrian University of Athens, 15701 Athens, Greece; Katia_13g@hotmail.com (A.F.G.); EKONSTANTAKOU@mgh.harvard.edu (E.G.K.); tveletz@biol.uoa.gr (A.D.V.); npapand@biol.uoa.gr (N.C.P.); skatarachia@biol.uoa.gr (S.K.); labant@aua.gr, (A.D.L.); veconom@biol.uoa.gr (V.I.); ipapasid@biol.uoa.gr (I.S.P.)
2 Laboratory of Molecular Carcinogenesis and Rare Disease Genetics, Institute of Biosciences and Applications, National Center for Scientific Research "Demokritos", 15310 Athens, Greece; savgeris@bio.demokritos.gr (S.N.A.); mvoutsin@bio.demokritos.gr (G.E.V.)
3 Section of Biochemistry and Molecular Biology, Department of Biology, School of Science, National and Kapodistrian University of Athens, 15701 Athens, Greece; margaritis.avgeris@gmail.com (M.A.); ascorilas@biol.uoa.gr (A.S.)
4 Department of Biological Chemistry, Medical School, National and Kapodistrian University of Athens, 11527 Athens, Greece; ilianna.zoi@gmail.com (I.Z.); cpiperi@otenet.gr (C.P.); ebasdra@med.uoa.gr (E.K.B.); papavas@med.uoa.gr (A.G.P.)
5 Center of Basic Research, Biomedical Research Foundation of the Academy of Athens, 11527 Athens, Greece; vickyrougefilippa@gmail.com (V.F.); synticha@bioacademy.gr (P.S.); anastasiadou@bioacademy.gr (E.A.)
6 Laboratory of Chronobiology, Institute of Biosciences and Applications, National Center for Scientific Research "Demokritos", 15310 Athens, Greece; prombona@bio.demokritos.gr
7 2nd Department of Dermatology and Venereology, Medical School, National and Kapodistrian University of Athens, "Attikon" University Hospital, 12462 Athens, Greece; epapad@med.uoa.gr
* Correspondence: dstravop@biol.uoa.gr; Tel.: +30-210-727-4105
† These authors contributed equally to this work.
‡ Current address: Massachusetts General Hospital Cancer Center, Harvard Medical School, Charlestown, Boston, MA 02114, USA

Supplementary Materials

Table S1. Gene name (symbol), Oligonucleotide primer sequence (F/R), Exon number (primer design position), Ensembl transcript number, Molecular size (bp) of PCR product, Annealing temperature (Ta) of primer and Number of cycles (PCR amplification program) for the herein examined genes are shown. F: forward (primer), R: reverse (primer) and bp: base pair

Gene	Primer	Sequence	Exon	Ensembl Transcript	bp	Ta (˚C)	Number of Cycles
CAMK1D	F	5'- AACATCCACGAGTCCGTCAG -3'	9	ENST00000619168.4	192	54	30
	R	5'- GGAAGGTGCCAGACAGTCTTT -3'	10_11				
C-MYC	F	5'- TTCTCTCCGTCCTCGGATTC -3'	2	ENST00000621592.5	188	57	32
	R	5'- TCTGACCTTTTGCCAGGAGC -3'	3				
GAPDH	F	5'- TGGTATCGTGGAAGGACTCAT -3'	7	ENST00000229239.9	189	55	28
	R	5'- ATGCCAGTGAGCTTCCCGTTCAGC -3'	8				
GMFG	F	5'- TCTGACTCCCTGGTGGTGTG -3'	2	ENST00000598034.5	237	54	30
	R	5'- GCCATCGTCATGCACGTACT -3'	5				
HGF	F	5'- CCTATTACGAGTGGCACATC -3'	17	ENST00000222390.9	216	55	35
	R	5'- ATGGCACATCCACGACCAG -3'	18				
MCT1	F	5'- TTGGAGGTCCAGTTGGATAC -3'	2	ENST00000538576.5	313	54	33
	R	5'- ACGGTGTTACAGAAAGAAGC -3'	3				
MCT4	F	5'- TGGTGGCTGCGTCCTTTTG -3'	2	ENST00000581287.5	178	56	33
	R	5'- CACAGGAAGACAGGGCTAC -3'	3				
MEOX2	F	5'- TCACCAGACTGAGGCGATAC -3'	2	ENST00000262041.5	147	54	30
	R	5'- TCACCAGTTCCTTTTCCCGAG -3'	3				
NOXA	F	5'- GTGCCCTTGGAAACGGAAGA -3'	2	ENST00000316660.6	258	55	35
	R	5'- CCAGCCGCCCAGTCTAATCA -3'	2				
PRR11	F	5'- CCGAAAGCACGGAATCCACT -3'	7	ENST00000262293.8	247	54	30

	R	5'- TCCTTAAGGCCTGCGTCATC -3'	9				
Sestrin-1	F	5'- CTTCTGGAGGCAGTTCAAGC -3'	9	ENST00000436639.6	341	57	30
	R	5'- TGAATGGCAGCCTGTCTTCAC -3'	10				
Sestrin-2	F	5'- CAAGCTCGGAATTAATGTGCC -3'	10	ENST00000253063.3	323	57	30
	R	5'- CTCACACCATTAAGCATGGAG -3'	10				
SRPX2	F	5'- CTGCCTATGACCGAGCCTAC -3'	7	ENST00000373004.4	178	56	30
	R	5'- GTCCCCTGGCGATCATAACC -3'	8				
Survivin	F	5'- GAGCTGCAGGTTCCTTATC -3'	5	ENST00000301633.8	433	53	30
	R	5'- ACAGCATCGAGCCAAGTCAT -3'	5				
SUSD6	F	5'- CCAGAGCGAGCTAGACAAGG -3'	1	ENST00000342745.4	241	54	30
	R	5'- ACTGAGGTGCTCTTTGGTGC -3'	2				
TSTD3	F	5'- AGAAGGAGTAGGAGGAAGTGGT -3'	1	ENST00000452647.2	172	55	30
	R	5'- AAACCTTCAGACCAGCCACAC -3'	2_3				
XIAP	F	5'- GAAGACCCTTGGGAACAACA -3'	3	ENST00000371199.7	225	55	32
	R	5'- GTCCTTGAAACTGAACCCCA -3'	6				

Table S2. Collection of gene transcripts being -potentially- targeted by more than three miRNA species that derive from the *c-MYC* (2/3), *Sestrin-1* (9/10) and *MCT4* (2/3) retained introns (also, see Fig. 4 and Table 1). Name (symbol) of target gene, Number of miRNA species per gene, Name of human miRNA molecule, Name (symbol) of gene being subjected to intron retention, Target score (success level of miRNA complementary binding to target transcript sequence) and Complete gene name (description) (reflects structural/functional protein's features) are described. hsa: *Homo sapiens*

Target Genes	Number of miRNAs	miRNAs	Intron Retained	Target Score	Complete Name
HGF	4	hsa-miR-5585-3p	*c-MYC* + *Sestrin-1*	52	Hepatocyte Growth Factor
		hsa-miR-4526	*MCT4*	54	
		hsa-miR-5096	*c-MYC* + *Sestrin-1*	71	
		hsa-miR-1273g-3p	*c-MYC* + *Sestrin-1*	89	
TSTD3	4	hsa-miR-5585-3p	*c-MYC* + *Sestrin-1*	50	Thiosulfate Sulfurtransferase (rhodanese)-like Domain Containing 3
		hsa-miR-5096	*c-MYC* + *Sestrin-1*	52	
		hsa-miR-619-5p	*c-MYC* + *Sestrin-1*	58	
		hsa-miR-5095	*Sestrin-1*	78	
PRR11	6	hsa-miR-1273g-3p	*c-MYC* + *Sestrin-1*	50	Proline Rich 11
		hsa-miR-3130-3p	*Sestrin-1*	55	
		hsa-miR-7851-3p	*c-MYC*	61	
		hsa-miR-619-5p	*c-MYC* + *Sestrin-1*	65	
		hsa-miR-5095	*Sestrin-1*	72	
		hsa-miR-5096	*c-MYC* + *Sestrin-1*	74	
CAMK1D	9	hsa-miR-767-5p	*MCT4*	53	Calcium/calmodulin Dependent Protein Kinase ID
		hsa-miR-5095	*Sestrin-1*	56	

		hsa-miR-548aj-3p	*c-MYC*	56	
		hsa-miR-548aq-3p	*c-MYC*	56	
		hsa-miR-1273f	*Sestrin-1*	60	
		hsa-miR-6795-3p	*MCT4*	61	
		hsa-miR-619-5p	*c-MYC + Sestrin-1*	81	
		hsa-miR-1273g-3p	*c-MYC + Sestrin-1*	87	
		hsa-miR-5096	*c-MYC + Sestrin-1*	94	
SUSD6	11	hsa-miR-1227-5p	*MCT4*	65	Sushi Domain Containing 6
		hsa-miR-3613-3p	*c-MYC*	65	
		hsa-miR-765	*MCT4*	66	
		hsa-miR-4526	*MCT4*	69	
		hsa-miR-619-5p	*c-MYC + Sestrin-1*	75	
		hsa-miR-1273g-3p	*c-MYC + Sestrin-1*	76	
		hsa-miR-548aj-5p	*c-MYC*	78	
		hsa-miR-548g-5p	*c-MYC*	78	
		hsa-miR-548x-5p	*c-MYC*	78	
		hsa-miR-5096	*c-MYC + Sestrin-1*	85	
		hsa-miR-149-3p	*MCT4*	98	

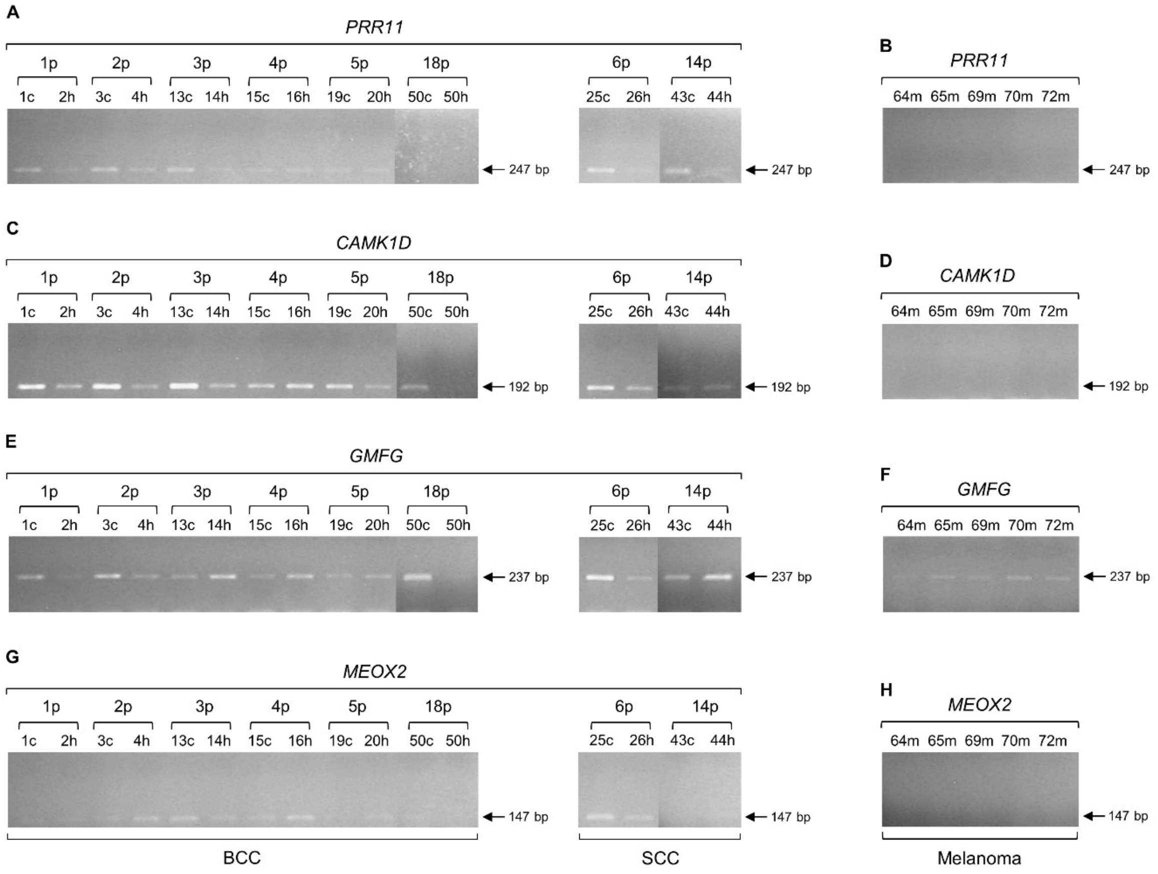

Figure S1. Melanoma-specific transcriptional repression of genes being targeted by intron-hosted miRNAs or encoding -cognate- protein interactome members. (**A**-**H**) Transcriptional expression profiles, via RT-sqPCR protocols employment, of intron-derived miRNA target genes (**A**-**D**) and genes responsible for the synthesis of members belonging to their respective protein interactomes (**E**-**H**) (also, see Fig. 4), in BCC and SCC (**A**, **C**, **E** and **G**), or melanoma (**B**, **D**, **F** and **H**) biopsy collections. (**A** and **B**) *PRR11* gene. (**C** and **D**) *CAMK1D* gene. (**E** and **F**) *GMFG* gene. (**G** and **H**) *MEOX2* gene. *GAPDH* was used as gene of reference (also, see Fig. 6F and Table S1). p: patient, c: cancer tissue (biopsy), h: healthy tissue (biopsy), m: melanoma (biopsy) and bp: base pair.

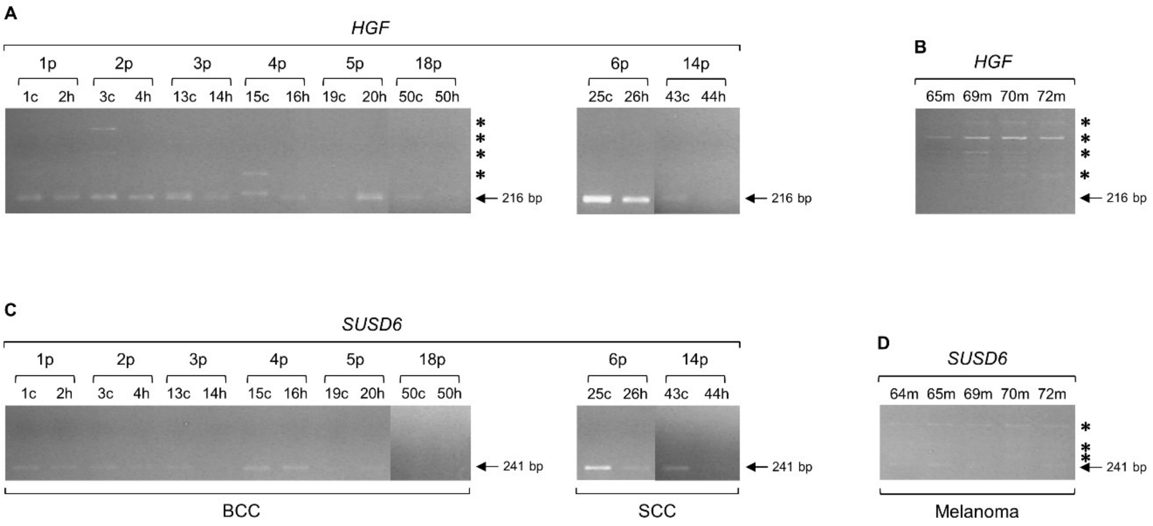

Figure S2. Gene-specific incidents of aberrant splicing in melanoma biopsies derived from Greek patients herein studied. Expression patterns of *HGF* (**A** and **B**) and *SUSD6* (**C** and **D**) genes, via employment of RT-sqPCR protocols, using total RNA preparations purified from BCC and SCC (**A** and **C**), or melanoma (**B** and **D**) cDNA collections. *GAPDH* served as gene of reference (also, see Fig. 6F and Table S1). p: patient, c: cancer tissue (biopsy), h: healthy tissue (biopsy), m: melanoma (biopsy), bp: base pair and asterisks (*): aberrant splicing-derived PCR fragments.

A

CA1ICL-861
MGGRGEELLTSQTGRLGRGVPHFPDGVAGQRHSSLPRRGGRAEALLTYQKGRPGRGAPHFPDGAAGQRRSSLPRWGGRAEVLFTSQM
MGGRAEALLTSHMMGGQADALLTSQKMGRRAEALLTSQTGQSGRGPPLFPDEAARQRRSSLLPRRGSRAEALPTSQKMGGRAEALLTS
QTMGGRAETIGGRAEALLTSQMGRPSRGAPHFSDWAAGPRRSSLPRQGGRAEAFLTSQTGXPGRGAPHFPEGVAGQRRSSLTRRGGR
AEALLTSQXRGGRAEALLTYQKGRPGRGAPHFPDGAAGQRCSSLPRWGGRAEVLFTSQMXGWPGRGTPHFPDGVAGQRHSSLPRRSV
ARQRPSSLPRWGGRAEALLTSQTGRPGRGAPHFPDGAAGQRLSSLPRHCGQAEALLISQTMGGQAEALLTSQTGRPGRGALHFPDGVA
GQRRSSLPRRGSWAEALPTSQMMGGQAEALLTSQMMGGQAEVLLTSQMMGGRADALLSSQMMGGRAEVLLTSQTERSGRGPPLFP
DEAAGHRRSSLLPRLGGRAEALLTSQTGWLGRGAPHFPDGAAGQRHSSFPRRWVARQRCSSLPRRGGRAEALLTSLTMGGRAEVLLTSQ
IIGGWAEALLTSQMGWTGRGAPHFPDDRWLGRGAPYFPDGAAGQRHSSLLPRWGDRAEVLLTSYQTGRPGRGAPHFPDGAAGQRRS
SLPRRGGRAEALLTSQTMGGQAEALLTSQTMGGQAEALLTSQTMCVGAEALLTSQMGRPSRGAPHFPDGAARQRCSSLPIRWVAGQK
RSSLPRRWVAVERRSSLPRRGSRAEALLTSQTGRPGRGAPHFPDGAAGQRRCPLPRRWVAGQRRSSLPRWGGQAEVL

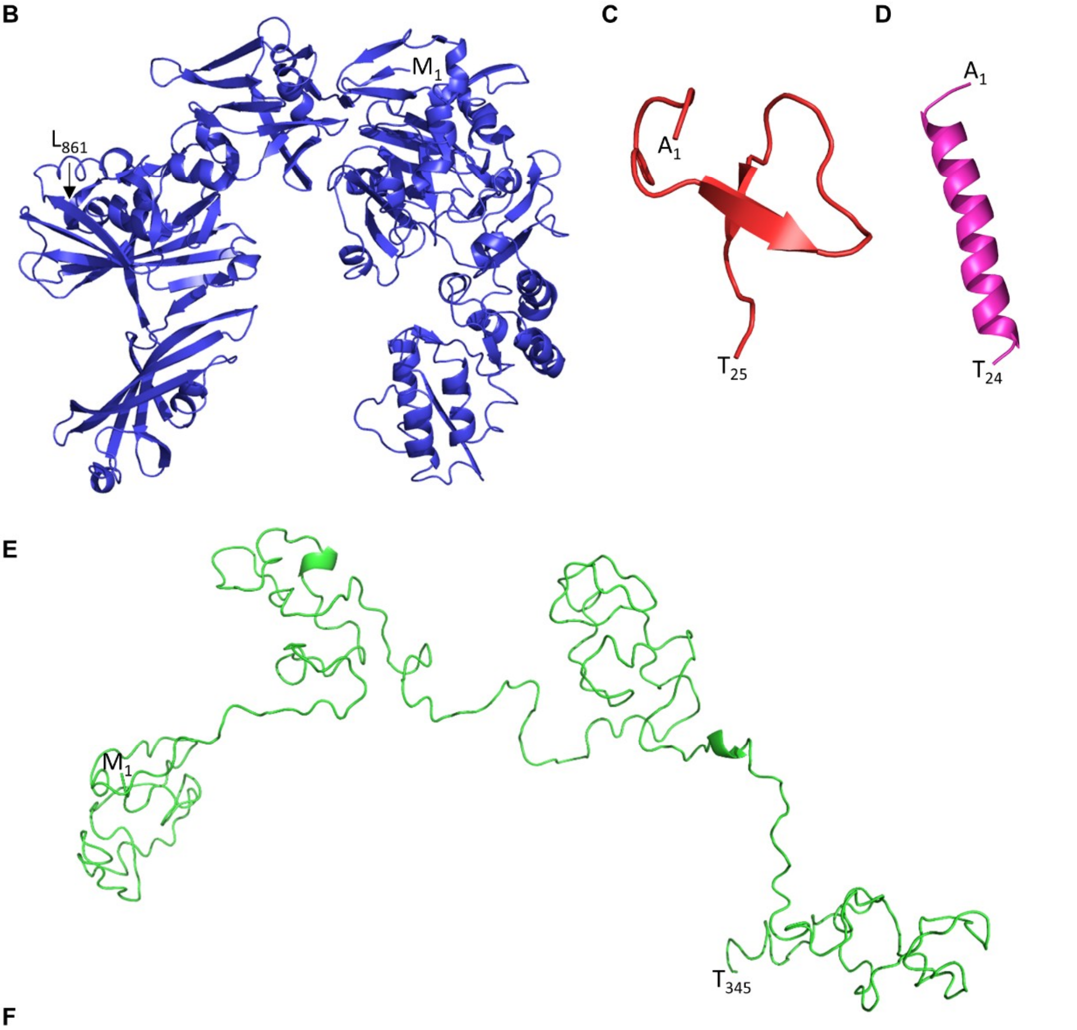

F

MODEL	C-score	TM-score	RMSD
CA1ICL-861	0.28	0.75 ± 0.10	7.9 ± 4.4 Å
ORF_SIN136 MOTIF AAGRRGSSLLIRGGCQAEGLLTSQT	-1.91	0.49 ± 0.15	5.0 ± 3.2 Å
ORF_SIN81 MOTIF AAGRRRSSLSRLGSQAEGLLTSQT	-0.63	0.63 ± 0.13	2.5 ± 1.8 Å
ORF_CIN345	-2.13	0.46 ± 0.15	11.5 ± 4.5 Å

Figure S3. Tertiary structure predictions of proteins carrying the "LLTSQ", or "STPSV" pentapeptide repeat. (**A**) Amino acid sequence of the CA1ICL-861 protein. The "LLTSQ" repeats are indicated by fonts with blue shading, while the "GRAE" tetrapeptide distinct repeats are also underlined. (**B**-**E**) 3D molecular models of selected proteins (**B** and **E**) and ORF motifs (**C** and **D**)

containing either the "LLTSQ" (**B**-**D**) or the "STPSV" (**E**) repeat, via engagement of I-TASSER bioinformatics algorithm. (**B**) CA1ICL-861 tertiary structure. (**C**) ORF_SIN136 motif ("AAGRRGSSLLIRGGCQAEGLLTSQT") tertiary structure. (**D**) ORF_SIN81 motif ("AAGRRRSSLSRLGSQAEGLLTSQT") tertiary structure. (**E**) ORF_CIN345 tertiary structure. The first (amino-terminal) (M_1 and A_1) and last (carboxy-terminal) (L_{861}, T_{25}, T_{24} and T_{345}) amino acid residues in each molecular structure are indicated. M: methionine, L: leucine, A: alanine and T: threonine. (**F**) Estimation of the quality and confidence to the herein predicted tertiary structure models (**B**-**E**), as evinced by the C-score, TM-score and RMSD values, respectively.

A

ORF_CIN345_modified
MVSVNTSSVTSVGTPSVSTGSVTSLSRPSVSSLSTGSVTSVSIPSVTSVGTPSVSTGSVTSVSTGSVTSVSTGSVTSVSTGSV
TSLSTGSVTSLSTGSVSTGSVPSLSTGSVTSLSTGSVTSLSTGSVTSVSTGSVTSLSTGSVSTGSVTSLSTGSVTSLSTGSVSTG
SVTSVRTPSVTSVSTGSVTSLSTGSVNTLSVASLSTGSVTSLGIPSVTSLSTGSVTSVSTGSVTTQYTISHISQYTISHITQYTIS
HISQYTISHISQYTSTGSVTSVSTGSVTSVSTGSVSTGSVTSLSTGSVTSVSTGSVISVSTGSVSTGSVTSLSTGSVQMDQES
MPHSLLNT

B

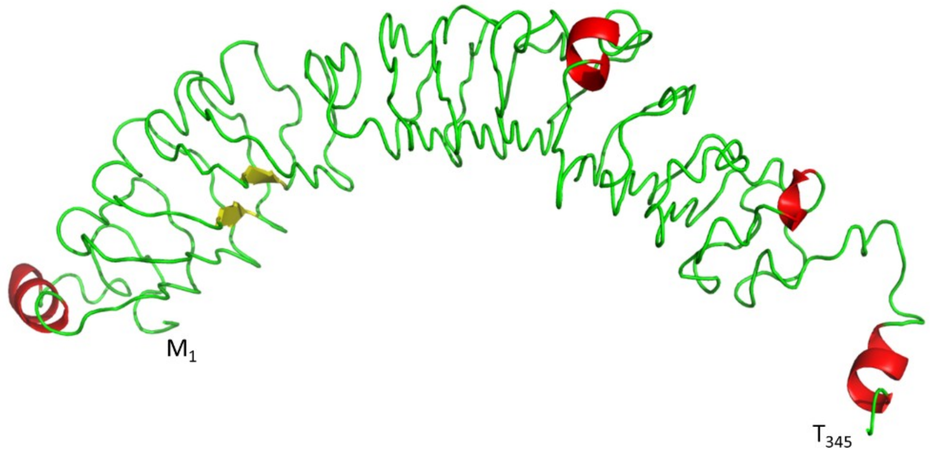

C

MODEL	C-score	TM-score	RMSD
ORF_CIN345_modified	-2.87	0.39 ± 0.13	13.4 ± 4.1 Å

Figure S4. (**A**) Amino acid sequence of the ORF_CIN345_modified protein. The *in silico* "mutated" "STGSV" ("G" has replaced "P" in each repeat) pentapeptide motif is indicated by fonts with blue shading. (**B**) 3D molecular model, via I-TASSER employment, of the ORF_CIN345_modified protein. Note the structural acquisition of alpha helices and beta strands, and the "spring"-like tertiary conformation of the *in silico* engineered protein. The first (amino-terminal) (M_1) and last (carboxy-terminal) (T_{345}) amino acid residues are indicated. M: methionine and T: threonine. (**C**) Estimation of the quality and confidence to the predicted tertiary structure model, as documented by the C-score, TM-score and RMSD values.
